# Architecting mechanosensitive nanofluidic transport in graphite nanoslits

**DOI:** 10.1126/sciadv.aed4451

**Published:** 2026-07-29

**Authors:** Mathieu Lizée, Zhijia Zhang, Baptiste Coquinot, Qian Yang, Lydéric Bocquet

**Affiliations:** ^1^ Laboratoire de Physique de l’Ecole Normale Supérieure, ENS, Université PSL, CNRS, 24 rue Lhomond, 75005 Paris, France.; ^2^ Department of Physics and Astronomy, University of Manchester, Manchester M13 9PL, UK.; ^3^ National Graphene Institute, University of Manchester, Manchester M13 9PL, UK.; ^4^ Institute of Science and Technology Austria (ISTA), Am Campus 1, 3400 Klosterneuburg, Austria.

## Abstract

Mechanosensitive ion transport plays a central role in enabling living systems to perceive and adapt to their environment through the deformation of soft, embedded ion channels. In this work, we demonstrate that ion transport within a two-dimensional graphite nanoslit can be rationally engineered to achieve a bipolar, pressure-sensitive response without any structural deformation. The mechanosensitivity arises from the selective charging of one channel inlet, which acts as a reversible source of mobile charge carriers. These excess ions can then be advected in or out of the channel by the pressure-driven water flow, thereby modulating the ionic conductance. This mechanism is captured through a comprehensive electrohydrodynamic model that analytically accounts for coupled diffusion, convection, surface transport, diffusio-osmosis, and interfacial slippage, both inside and outside the nanoslit. The theoretical framework quantitatively reproduces the experimental data, showing that a simple surface charge pattern can give rise to complex, pressure-dependent conductance. These findings reveal how rich nonlinear couplings at the nanoscale can be harnessed to design adaptive, bioinspired nanofluidic systems, exemplified here by ionic pressure sensors.

## Teaser

Local surface charge engineering enables ionic pressure sensing in graphitic nanofluidic channels.

## INTRODUCTION

Living organisms experience a great variety of mechanical forces to which they adapt. Consequently, nature has developed exquisite pressure sensors in the form of embedded ion channels across the cellular membrane that deforms under membrane tension or differential pressure drops producing strong current signals for pressure changes as low as 10 mbar (*1*, *2*). In parallel to the biological porins, solid-state nanopores and nanochannels offer new model systems with a high degree of structural and chemical control. In some nanopore geometries, strain-induced mechanical transduction was shown to mimic some pressure sensitivity (*3*, *4*).

However, beyond direct mechanical mimicry, the rich and intertwined transport of ions and fluids inside nanochannels offers a wealth of possibilities to modulate ion conduction under various external stimuli, allowing us to design sensing functions similar to those found in nature, albeit based on distinct working principles. Several experiments have reported nonlinear coupled transport effects, taking the form of pressure modulation of the conductance or voltage modulation of the streaming current (*5*–*9*). Depending on the geometry, these coupled behaviors were attributed to the modulation of the charge polarization in conical nanopores (*5*, *6*, *10*) or to the advection of potential-induced charges by the flow in carbon nanotubes or across graphene nanopores (*8*, *9*). Although it is now clear that mechanosensitivity can emerge via the coupling between ionic and hydrodynamic flow in the absence of mechanical deformation, the lack of a clear mechanism and suitable design hinders progress toward strain-free nanofluidic pressure sensing. Furthermore, while, in most of the aforementioned studies, the effects of pressure were symmetric under pressure reversal (ΔP→−ΔP), it would be desirable to design systems that are able to sense the sign of the pressure drop as well.

In this work, we demonstrate that a strong mechanosensitive behavior of a graphitic nanochannel can be engineered through the deliberate design of surface charge patterns, giving rise to a nonlinear pressure-dependent ionic transport. Our devices demonstrate a bipolar mechanosensitivity of the ionic conductance to pressure drops, which changes sign upon pressure drop reversal. The sensitivity reaches the 10-mbar range on a wide range of salt concentration and nanochannel height. The detailed modeling of the electrohydrodynamic transport across the nanochannel allows rationalizing the pressure-dependent response of the conductance. This behavior is shown to be rooted in the advection of the electrical double layer (EDL) by water flow induced by the selective charging of one channel’s inlet. We show that the observed mechanosensitivity is strongly enhanced by the low friction of water on graphite.

This work demonstrates a method for engineering artificial nanofluidic channels, inspired by the precise arrangement of charged groups and binding patches that are instrumental to the selectivity of biological porins (*11*, *12*). This mechanism could be used to transduce the ultralow mass flows in nanotubes or biological porins into electrical signals, allowing us to extract their permeability. Integrated into a small deformable cell, the device could also operate as a bidirectional nanofluidic pressure sensor, detecting millibar-range pressure drops through a simple electrical readout and without structural deformation.

## RESULTS

### Nanochannel fabrication

**Fig. 1.**
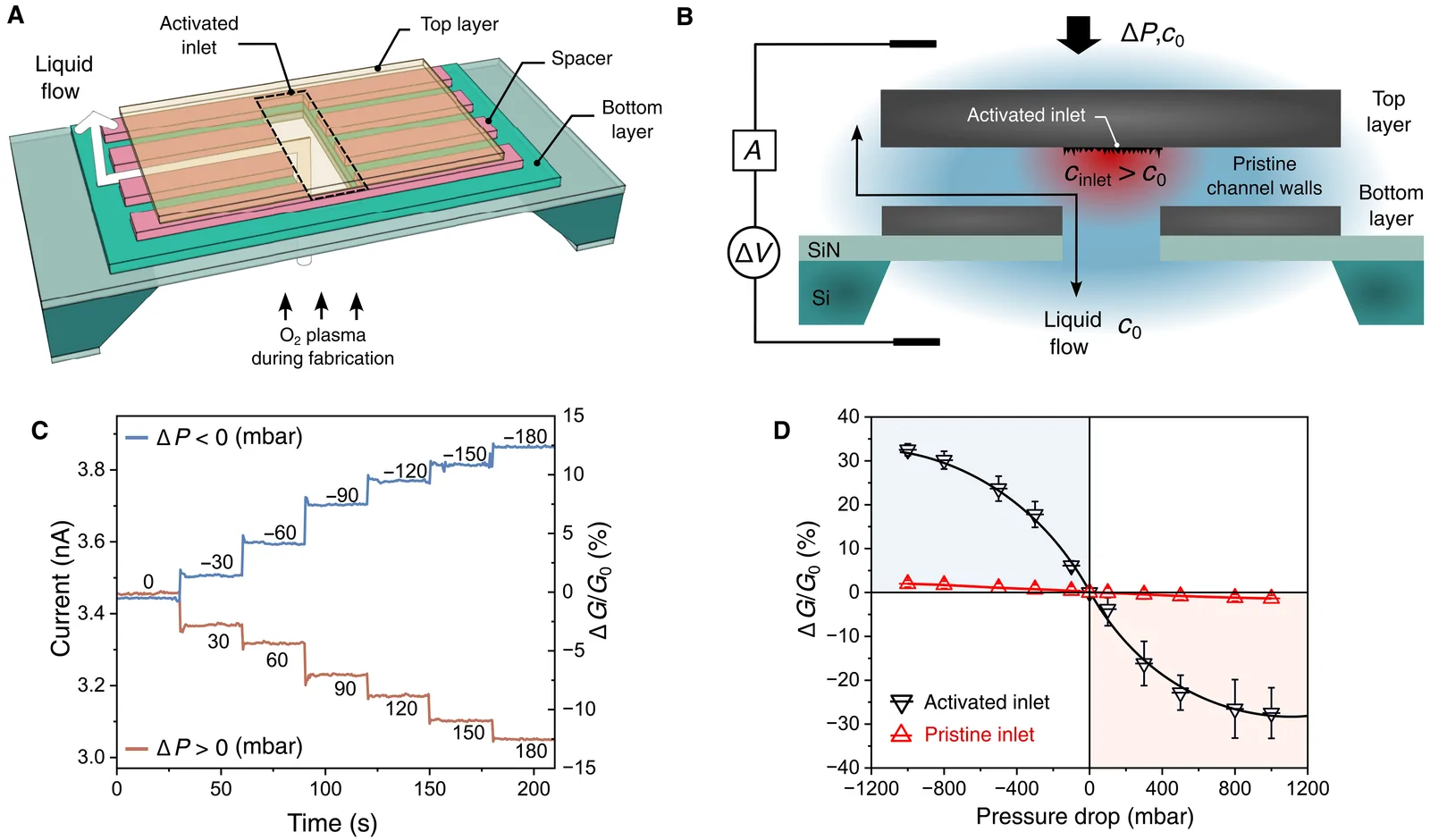
Mechanosensitive nanochannels experimental setup. (**A** and **B**) A 2D graphite nanochannel fabricated by van der Waals assembly is placed between two reservoirs filled with aqueous KCl solution. A voltage drop is applied between two Ag/AgCl electrodes placed in two reservoirs on either side of the channel. A pressure drop is also applied between the two reservoirs. By convention, the pressure drop is positive when the pressure is applied on the top side of the nanochannel, as indicated by the arrow on top. (**C**) The measured current from a 1-nm activated channel in 1 M KCl solution under both positive and negative pressure drop steps and a voltage drop Δ*V* = 100 mV. (**D**) The relative conductance change $\Delta G/G_{0}$ as a function of applied pressure for both plasma-activated channels (*h* = 1 nm; black symbols) and pristine channels (*h* = 1.3 nm; red symbols) in 1 M KCl solution. Solid lines are guide for the eyes.

Two-dimensional (2D) graphite slits are fabricated by van der Waals assembly following the technique introduced in (*13*) (see Fig. 1A and details in note S1). Briefly, the channels were made of multilayer graphite spacers sandwiched between bottom and top graphite crystals. The sandwiched assembly sits on a suspended silicon nitride (SiN) membrane with a rectangular opening, allowing ion transport (the white arrow in Fig. 1A indicates the ion transport pathway). The spacer layer was patterned using electron beam lithography (EBL) and reactive ion etching (RIE) to form tens of strips in parallel, which act as the side walls of the channels and define their height.

At the end of the fabrication process, we exposed the backside of the device to a mild Ar/O_2_ plasma (as shown by the black arrows in Fig. 1A) to create a patch of “activated carbon” at the bottom-side inlet of the channels. When immersed in water, such an activation generates a strong surface charge on the surface of graphite as compared to pristine graphite (*14*). As a control, we also produced “pristine-inlet” samples prepared in the exact same way except for the activation step. We show in the following that this selective activation is instrumental to the pressure sensing capability of our channels.

### Ion transport measurement

We measured the ionic transport properties of the channels by placing the device in a fluidic cell between two tightly bound reservoirs, which are filled with potassium chloride (KCl) aqueous solutions. A bias voltage ΔV and pressure drop ΔP can be applied across the nanochannel between the two reservoirs. All experiments presented here were performed at room temperature. To benchmark the experimental procedure, we first characterized the bare (ΔP=0) conductance of the channels at different concentrations, as well as the ionic current under voltage and concentration drops, respectively (see note S1). These results show excellent agreement with the preexisting body of experimental work on 2D graphite channels (*7*, *15*–*17*).

Turning now to the combined ΔV−ΔP drivings, we report in Fig. 1C the time traces of ionic current measured as a function of pressure under a 0.1-V bias in a 1-nm-height channel. We observe a clear drop of current in steps mirroring the pressure steps at positive ΔP (pressure applied from the topside of the channels) and, vice versa, an increase in current at negative ΔP, when pressure is applied from the backside of the channels. The measured current-voltage (*I*-*V*) response is linear at all pressures (see fig. S4), suggesting that the system is characterized by a pressure-dependent conductance $G\left(\Delta P\right)=I_{e}/\Delta V$ (with *I*_e_ denoting the measured ionic current under the voltage ΔV and pressure drop ΔP).

Let us note that throughout all measurements, the streaming current, induced by the advection of EDLs by the water flow and measured at zero voltage drop $I_{\text{str}}=I\left(\Delta P,\Delta V=0\right)$, remains in the tens of picoampere range (see figs. S3 and S6), while the channels’ conductance is of a few tens of nanosiemens. Therefore, for any bias voltage above a few millivolts, the streaming contribution is negligible.

In Fig. 1D, we plot the relative mechanoconductance $\Delta G/G_{0}$, where $G_{0}=G\left(\Delta P=0\right)$ and $\Delta G=G\left(\Delta P\right)-G_{0}$ of 1-nm-height channels in 1 M KCl solution as a function of pressure ΔP. We compare results from both activated-inlet channels (black symbols) and pristine-inlet channels (no plasma exposure; red symbols). Despite similar channel height (*h*), we observe strongly contrasting trends. The activated device, which received a post–plasma treatment of the inlet, shows a strong sigmoid-shaped modulation of the conductance, with a relative increase exceeding 30%, and a saturation of the pressure modulation above 500 mbar. In contrast, for channels with pristine, nonactivated inlets, a much smaller modulation—less than 2% at 1 bar—is observed. Notably, the sign of the pressure drop directly determines that of the conductance change. That is, conductance drops when pressure is applied on the top side of the chip, whereas it increases when pressure is applied on the back, yielding an odd response to pressure drops [ΔG(−ΔP)=−ΔG(ΔP)], which is rarely observed in mechanosensitive ion channels.

### Ruling out mechanical deformations

We now demonstrate that the observed mechanosensitivity in our geometry can be explained by the nonlinear couplings between ionic conduction and fluid transport, which are enhanced by the surface charge pattern characteristic of the present system. However, before entering into the ion dynamics, let us first show that mechanical deformations can be ruled out as origins to the pressure-sensing behavior of our channels. Such a mechanism was invoked as a source of mechanosensitivity in some artificial solid-state systems (*3*, *4*), and we first estimate the mechanical deformations of the channel walls and the SiN membrane.

Under a pressure drop ΔP, the sagging of the graphite top layer forming the nanochannel (with thickness H=50 nm, channel’s width w=200 nm, and Young’s Modulus E=1 TPa) reads $\delta =5\Delta {\mathrm{Pw}}^{4}/32{\mathrm{EH}}^{3}$ (*18*, *19*). For ΔP as high as 1 bar, we find a deformation of δ∼0.2 pm, ruling out any measurable effect of the deformation of the channel wall on the conductance. We also ruled out the deformation of the SiN support membrane as a source of pressure-dependent conductance. To check this effect, we fabricated a 2.4-nm-high channel on a much thinner SiN membrane (100 nm instead of 500 nm). We show in fig. S5 that the mechanosensitivity in these devices is similar for the 100- and 500-nm-thick SiN membranes despite a 125-fold difference in the stiffness of the SiN membrane.

### The activated inlet as a source of disposable charge carriers: A 1D advection model

The absence of mechanical effect thus suggests an electrohydrodynamic origin for the mechanosensitivity. The mechanosensitive response observed here is, however, peculiar as compared to some previous observations. First, the pressure response reported here is odd with respect to ΔP:ΔG(−ΔP)=−ΔG(ΔP), in contrast to the symmetric dependence in carbon nanotubes and across graphene nanopores reported before (*8*, *9*). Furthermore, we invariably measured a perfectly linear current-bias response at all pressure drops (see fig. S4), and this rules out mechanisms that are intrinsically nonlinear in ΔV (*5*, *9*, *10*). This implies that the present phenomenon originates from a distinct set of underlying physical mechanisms.

A crucial observation in our experiments is the role of the (oxygen-plasma) activation of the channel inlet, which considerably amplifies the pressure response of the conductance. The activation induces a strong surface charging of the graphite surface in water, typically of the order of several hundred millicoulombs per square meter (*14*), serving as a high surface charge patch at the bottom inlet (Fig. 1, A and B), while the channel itself remains pristine. This yields a strong surface charge asymmetry between the two inlets of the nanochannel, analog to systems previously investigated numerically (*20*).

#### General mechanism and transport framework

**Fig. 2.**
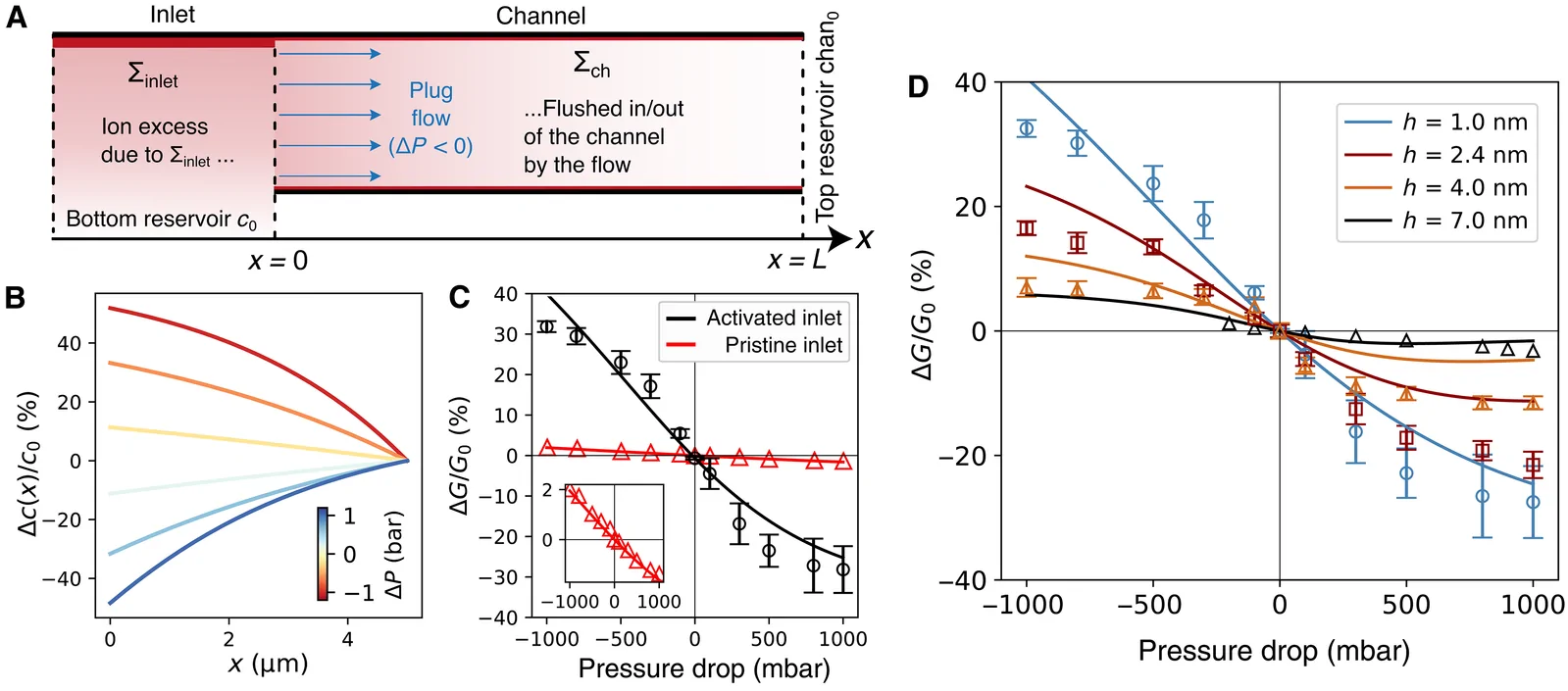
An electrohydrodynamic model for mechanosensitivity. (**A**) Sketch of the model: Countercharges near the activated inlet are advected by the water flow with applied pressure, resulting in a pressure-dependent concentration profile in the channel. Darker color represents higher concentration. (**B**) Theoretical predictions for the pressure-dependent relative concentration drop profiles in a 1-nm-height channel plotted for *h* = 1 nm, *L* = 5 μm, *b* = 20 nm, $\Sigma_{\text{ch}}=-25\;\text{mC}/m^{2}$, and $\Sigma_{\text{inlet}}=-150\;\text{mC}/m^{2}$ at $c_{0}=1\;M$, closely matching experimental conditions. (**C**) Experimental results for the mechanoconductance of 1-nm-height channels with respectively a pristine (red) and an activated inlet (black) versus the theoretical predictions: Solid lines are one-parameter fits with the model, with the slip length *b* fixed at 20 nm, the channel surface charge density $\Sigma_{\text{ch}}=-25\;\text{mC}/m^{2}$, and the inlet’s surface charge density $\Sigma_{\text{inlet}}$ as a single free parameter. From the fit of the activated-inlet (black curve) and pristine-inlet (red curve) data, we respectively obtain $\Sigma_{\text{inlet}}=-204\pm 10\;\text{mC}/m^{2}$ for activated channels and $\Sigma_{\text{inlet}}=-50\pm 1\;\text{mC}/m^{2}$ for pristine-inlet channels. (**D**) Mechanoconductance measurements at *c*_0_ = 1 M on the whole pressure range between −1 and 1 bar for h∈{1,2.4,4,7} nm against the theoretical predictions. Fit parameters are, respectively, $\Sigma_{\text{inlet}}\in \left\{-204,-160,-127,-102\right\}\pm 10\;\text{mC}/m^{2}$, *b* = 20 nm, and $\Sigma_{\text{ch}}=-25\;\text{mC}/m^{2}$.

Starting from the Poisson-Nernst-Planck-Stokes (PNPS) framework, the following mechanism emerges from the solution of the transport equations: The ions in the EDL close to the activated surface are flushed toward (or away from) the channel by the water flow. The surface charge discontinuity at the channel inlet leads to a pressure-dependent salt concentration profile in the nanochannel (see Fig. 2, A and B). The local ionic conductance, which is proportional to the concentration of free ions in the channel, increases for negative pressure drops, which flush the inlet concentration polarization into the channel (as sketched on Fig. 2A), and decreases for positive pressure drops, which flush it out of the channel.

#### Capillary pore model and scale separation

While the scenario is relatively simple, the detailed mechanism combines diffusion, convection, surface conduction, and diffusio-osmotic (DO) transport, making the implementation of the model quite involved. However, it can be simplified to obtain analytical predictions for the ion conduction, amenable to direct comparison with the experiments.

To simplify the calculations while keeping the main physical ingredients, we reduced the geometry to that of a continuous slit, with height *h*, length *L*, and surface charge $\Sigma_{\text{ch}}$ connected to the activated inlet, which we modeled as a channel of height *h* and length $L_{1}$ whose walls bear a charge $\Sigma_{\text{inlet}}$ (see note S2). Overall, this simplified geometry retains the charge heterogeneity and the feeding of the nanochannel by the charges within the EDL at the inlet’s surface as the main ingredient for the observed behavior. We further validated this simplified assumption by comparing our analytical predictions to exhaustive finite element calculations in a geometry, which very closely reproduces experimental conditions. All details are given in note S3. The description then follows the capillary pore (or space charge) model discussed in (*21*). Under a pressure drop ΔP, the averaged water velocity in the channel is given by $v_{w}=\Delta P\frac{h^{2}}{12\eta L}\left(1+6b/h\right)$, where h and L are channel’s height and length, respectively, b is the slip length, and η is water’s dynamic viscosity. We introduce the Péclet number $\text{Pe}=v_{w}L/D$ comparing the advection at scale L to diffusion (with D the diffusion coefficient)

$$\text{Pe}=\frac{\mathrm{hb}\Delta P}{2\eta D}$$ (1)

where we assume b≫h in the nanochannel, an assumption that will be validated below. Turning now to ionic transport, we focus on the limit where the Debye length is smaller than the channel height, which is relevant under our experimental conditions. We accordingly use the scale separation methodology introduced by Poggioli *et al.* (*22*) to rewrite the ionic transport equations by integrating transport over the EDL contributions. Details are given in note S2.

Taking into account the diffusion, surface contributions, and convection, the total ion flux of both cations and anions, J, is calculated from the salt concentration profile c(x) (the sum of cation and anion concentration profiles) as

$$J=-AD\left(1+F\left[\text{Du}\left(x\right)\right]\right)\left(\partial_{x}c\left(x\right)-\text{Pe}\;\frac{c\left(x\right)}{L}\right)$$ (2)

where A is the channel’s cross section, and we introduced the local Dukhin number Du(x)=Σ(x)/hc(x) accounting for the ratio of surface charge Σ to bulk ion content hc(x). The term F[Du(x)] accounts for the surface contribution to transport within the EDL and is calculated as

$$F\left[\text{Du}\right]=2\frac{\lambda_{D}}{h}\left(\sqrt{1+{\left[\frac{h\text{Du}}{2\lambda_{D}}\right]}^{2}}-1\right)$$ (3)

where $\lambda_{D}=\sqrt{\epsilon k_{B}T/2e^{2}c}$ is the Debye length.

Equation 2 can be solved for the concentration profile c(x) inside the nanochannel and in the inlet region. As boundary conditions, c(x) asymptotically matches the bulk ion concentration $c_{0}$ in both reservoirs at x→±∞. Furthermore, we match concentration profiles and ion fluxes between the inlet region and the channel. Results are analytical for low Péclet number. For high Péclet number, the 1D differential equation is then solved numerically to obtain the concentration profile c(x) and ion flux J in the nanochannel. Last, from the salt concentration profile, we compute the conductance from the local Ohm’s law

$$G∼1/\int_{0}^{L}{\left[c\left(x\right)+\Sigma_{\text{ch}}/h\right]}^{-1}\mathrm{dx}$$ (4)

and the relative mechanosensitivity $\Delta G/G_{0}$, which directly compares to experiments.

### DO correction

Now, under the flow-induced concentration gradient, a DO flow builds up and adds to the bare pressure-driven flow, echoing experimental findings in carbon nanotubes (*23*). The DO velocity takes the form $v_{\text{DO}}=K_{\text{DO}}\Delta c\left(x=0\right)/L$, where the DO mobility can be estimated as (*24*)

$$K_{\text{DO}}≃\frac{k_{B}T}{\eta }\lambda_{D}^{2}\left(1+\frac{b}{\lambda_{D}}\right)\approx \frac{k_{B}T}{\eta }\lambda_{D}b$$ (5)

with $k_{B}T$ representing the thermal energy, $\lambda_{D}$ representing the Debye length, and $b\gg \lambda_{D}$ representing the slip length. To compute $v_{\text{DO}}$, we estimate the concentration drop across the channel at first order in ΔP.

At low-pressure drops (Pe≪1), one may deduce analytically the excess concentration at the entrance of the channel as

$$\frac{\Delta c\left(x=0\right)}{c_{0}}=\Gamma \times \text{Pe}$$ (6)

with Γ being a function of the Dukhin numbers associated with the channel (${\text{Du}}_{\text{ch}}$) and activated patch (${\text{Du}}_{\text{inlet}}$) surface charge

$$\Gamma =\frac{F\left[{\text{Du}}_{\text{inlet}}\right]-F\left[{\text{Du}}_{\text{ch}}\right]}{2+F\left[{\text{Du}}_{\text{inlet}}\right]+F\left[{\text{Du}}_{\text{ch}}\right]}$$ (7)

Now, since the concentration drop is induced by the flow itself $\Delta c\left(x=0\right)=c_{0}\;\Gamma \;\text{Pe}\propto v_{w}$, one deduces that the DO velocity is proportional to the pressure-driven water velocity in the form $v_{\text{DO}}=c_{0}\frac{k_{B}T}{\mathrm{\eta D}}\lambda_{D}\;b\times \Gamma \times v_{w}$. The total water velocity is now $v_{\text{eff}}=v_{w}+v_{\text{DO}}=v_{w}\times \left(1+c_{0}\frac{k_{B}T}{\mathrm{\eta D}}\lambda_{D}\;b\;\Gamma \right)$. Accordingly DO transport renormalizes the Péclet number as

$${\text{Pe}}_{\text{eff}}=\text{Pe}\left[1+c_{0}\frac{k_{B}T}{\mathrm{\eta D}}\lambda_{D}\;b\;\Gamma \right]$$ (8)

where the coefficient Γ is defined in Eq. 7. Because of the slippage effect, this correction to the Péclet number is large, and hydrodynamic slip acts as a booster of the mechanosensitive effect.

### Low pressure-drop limit

In the low pressure-drop limit, the relative mechanoconductance $\Delta G/G_{0}$ takes the final expression

$$\frac{\Delta G}{G_{0}}=\frac{1}{1+{\text{Du}}_{\text{ch}}}\cdot \frac{F\left[{\text{Du}}_{\text{inlet}}\right]-F\left[{\text{Du}}_{\text{ch}}\right]}{2+F\left[{\text{Du}}_{\text{inlet}}\right]+F\left[{\text{Du}}_{\text{ch}}\right]}\times \frac{{\text{Pe}}_{\text{eff}}}{2}$$ (9)

which scales linearly in the pressure drop ΔP (see note S2). The response is shown to vanish in the absence of charge contrast (when $\Sigma_{\text{inlet}}=\Sigma_{\text{ch}}$). This result therefore highlights the key role of the charge pattern in the mechanosensitive response, in agreement with the experimental observations (see Fig. 1D). The prediction in Eq. 9 shows a nontrivial dependence of mechanosensitivity on the bulk salt concentration $c_{0}$, as we now discuss.

#### High-concentration regime

First, at high salt concentration, surface contributions become negligible as the Dukhin numbers vanish and $F\left[\text{Du}\right]≃\frac{h}{4\lambda_{D}}{\text{Du}}^{2}\to 0$. Further, assuming that $\Sigma_{\text{inlet}}\gg \Sigma_{\text{ch}}$ due to plasma activation, the Péclet dependence of the conductance then reduces to the expression

$$\frac{\Delta G}{G_{0}}\approx \frac{1}{16h\lambda_{D}c_{0}^{2}}\frac{\Sigma_{\text{inlet}}^{2}}{4}\times {\text{Pe}}_{\text{eff}}$$ (10)

The relative mechanoconductance $\Delta G/G_{0}$ scales as $1/\left(\lambda_{D}c_{0}^{2}\right)∼c_{0}^{-3/2}$, dropping at high concentration. If the surface charge is regulated by salt concentration with a power α ($\Sigma_{\text{inlet}}∼c_{0}^{\alpha }$) as previously reported on carbon surfaces (*14*, *25*), then the decay at high concentration is weaker, scaling as $∼c_{0}^{-3/2+2\alpha }$.

#### Low-concentration regime

At low salt concentration on the other hand, the Dukhin number is large, so that F[Du]≃Du. Again, assuming that $\Sigma_{\text{inlet}}\gg \Sigma_{\text{ch}}$, we find

$$\frac{\Delta G}{G_{0}}\approx \frac{1}{2{\text{Du}}_{\text{ch}}}\times {\text{Pe}}_{\text{eff}}$$ (11)

Equation 11 predicts that mechanosensitivity vanishes linearly at low salt concentration $c_{0}$. This results from the dominating surface conductance in the channel at high Dukhin number.

Together, this analysis suggests that mechanosensitivity vanishes both at high and low concentration, thus resulting in a nonmonotonic variation with concentration. We will test this prediction against experimental results in the following section.

### General solution

#### Concentration profile

Beyond the low limit, the EDL-advection model in Eq. 2 is solved numerically to obtain the concentration profiles c(x) inside the nanochannel and in the inlet region (see Fig. 2B and note S2). On Fig. 2B, we plot the differential concentration profiles $\Delta c\left(x\right)/c_{0}$ for a given set of parameters, which closely match the experimental conditions (see below). The profile shapes illustrate the diffusion-advection balance under increasing flushing effects at high Péclet.

#### Mechanosensitivity

We report the relative mechanoconductance $\Delta G/G_{0}$—computed from the c(x) profile with the local Ohm’s law (Eq. 4)—in fig. S11 for *h* = 1 nm, *L* = 5 μm, $\Sigma_{\text{ch}}=-25\;\text{mC}/m^{2}$, and $\Sigma_{\text{inlet}}=-150\;\text{mC}/m^{2}$ for a range of slip lengths and salt concentrations. $\Delta G/G_{0}$ is found to be an odd function of pressure, with a magnitude of the order of a few tens of percent for b∼20 nm, and is linear in ΔP at low Péclet number. The results also highlight a saturation at high ΔP, which we find to be slightly asymmetric—stronger for positive pressure drops. In fig. S11, we also plot the prediction for $\Delta G/G_{0}$ versus $c_{0}$ at a fixed slip length b=20 nm for a range of slit heights. As anticipated, we find that $|\Delta G/G_{0}|$ is suppressed at both high and low concentrations. The optimum concentration $c_{0}^{\text{max}}$, at which $|\Delta G/G_{0}|$ is maximum, shifts downward with increasing slit height, from roughly 1 M at h=1 nm to 200 mM at h=4 nm.

### Finite element calculations

To support the predictions of the 1D EDL-advection model, we performed finite element calculations of transport in the original geometry of the system, accounting precisely for the converging flow at the inlet-nanochannel interface and for charge separation regions. Using COMSOL, we solved accordingly the complete set of nonlinear PNPS equations for the 2D geometry under consideration and explored the pressure dependence of the concentration profile, as well as the change in conductance. All details are provided in note S3. Solving this finite element model numerically, we find a pressure-dependent concentration profile in the channel, consistent with findings from the EDL-advection model (see figs. S13 to S16).

Using the finite element calculations, we checked that electric fields along x decay over less than 1 nm and that neglecting charge separation in the analytical model (Eq. 2) has little effect on the resulting conductance (see figs. S13 and S14). The numerical results fully reproduce the odd pressure response at low ΔP, the asymmetric saturation at high ΔP, and the order of magnitude of the $\Delta G/G_{0}$ (see fig. S15). We also recover the scaling $\Delta G/G_{0}∼\Sigma_{\text{inlet}}^{2}$ predicted in the low–Dukhin number limit (see Eq. 10 and fig. S16). Overall, the finite element calculations support the hypothesis made and confirm the predictions of the 1D EDL-advection model. We now use the predictions to fit the experimental data.

## DISCUSSION

We discussed above that the theoretical predictions successfully reproduce the overall experimental observations and trends: an odd mechanosensitive effect for the conductance and the saturation at large pressure. To fully characterize the pressure-sensing mechanism, we further examined experimentally the respective roles of confinement and salt concentration on mechanosensitivity. We accordingly investigated the properties of graphite channels with various heights h∈{1,2.4,4,7} nm, which were all subject to inlet activation by plasma treatment following the procedure described above.

Let us now compare systematically the predictions of the model with the experimental results. The model depends on several surface properties that are difficult to estimate independently: the surface charge of the channel and inlet and the slip length of the channel walls. To account for our measurements, we fix the surface charge $\Sigma_{\text{ch}}$ and slip length b at the channel walls for all channels and salt concentrations. The slip length is fixed at *b* = 20 nm to account for G(ΔP) measurements under imposed concentration drops in the pristine-inlet channels (see note S1E). The value is also close to direct measurements for pure water on graphite (8 nm) (*26*). The wall’s surface charge, on the other hand, is fixed at $\Sigma_{\text{ch}}=-25\;\text{mC}/m^{2}$ for a 1 M KCl solution and assumed to scale as $c_{0}^{1/3}$ with salt concentration, a regulation behavior reported in previous work (*15*, *25*). As a consequence, we leave the inlet charge $\Sigma_{\text{inlet}}$ as the only free parameter in the subsequent analysis.

### Comparison of pristine and activated inlets

The predictions for the pristine and activated inlets are compared in Fig. 2C with the experimental results at 1 M—the solid line is a fit taking $\Sigma_{\text{inlet}}$ as the only fitting parameter. The theoretical prediction successfully describes the pressure variation, and the difference in magnitude of the mechanosensitive effect is accounted for by using different surface charges on the inlet surface. Fitting the results under the assumption that *b* = 20 nm and $\Sigma_{\text{ch}}=-25\;\text{mC}/m^{2}$, we obtained values of $\Sigma_{\text{inlet}}=-50\pm 1$ and −204 ± 10 mC/m^2^ for the surface charge on the pristine-inlet and activated-inlet channels, respectively. Note that the concentration profiles in Fig. 2B were obtained with the parameters fitted to the activated-inlet channel data.

### Influence of channel height

The influence of the channel height is reported in Fig. 2D, where we plot the pressure-dependent mechanoconductance measured at 1 M concentration in channels of various heights h∈{1,2.4,4,7} nm (all are activated at the inlet). Here, we fit our model to the experimental data with the inlet charge as the only free parameter (here, again, *b* = 20 nm and $\Sigma_{\text{ch}}=-25\;\text{mC}/m^{2}$). Our model is shown to reproduce the experimentally observed mechanoconductance dependence for all confinement sizes *h*, with inlet charges changing in a relatively small interval, between −100 and −200 mC/m^2^. In particular, it accounts quantitatively for the sigmoid-shaped relative mechanoconductance and its suppression as the channel’s height increases, reducing from ∼30% at 1 nm down to a few percent at 7 nm.

### Surface charge regulation

We showed above that the model predicts the suppression of the mechanosensitive response for both vanishing and high salt concentrations. However, a quantitative investigation of the effect of salt concentration requires allowing for variations of the inlet surface charge with salinity $\Sigma_{\text{inlet}}\left(c_{0}\right)$ due to a possible surface charge regulation (*25*).

**Fig. 3.**
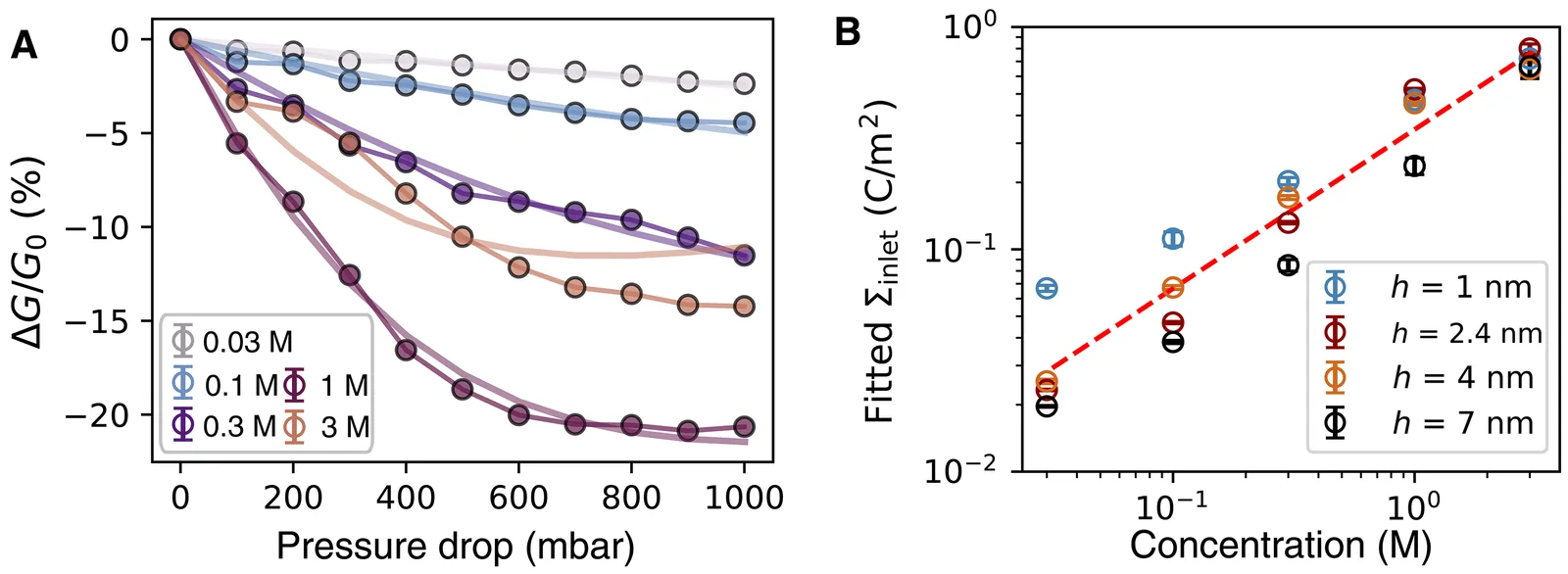
Concentration dependency of the mechanosensitive effect and fits with the theoretical model. (**A**) Mechanoconductance versus pressure curves for a channel with height *h* = 2.4 nm for ΔP ranging between 0 and 1 bar. Solid lines are a one-parameter fit with the theoretical model, assuming a slip length of 20 nm, a pristine graphite surface charge of −25 mC/m^2^ at *c*_0_ = 1 M, and a regulation of $\Sigma_{\text{ch}}∼c_{0}^{1/3}$ consistent with existing measurements on carbon nanotubes (*25*). (**B**) Surface charge $\Sigma_{\text{inlet}}$ for each ion concentration extracted from the fits of the theoretical predictions for various channel height (see fig. S9). We find that the data for all confinements collapse on a power-law dependence, $\Sigma_{\text{inlet}}∼c_{0}^{\alpha }$, with α=0.71±0.05 and independent of channel height (see the red dashed line).

We report in Fig. 3A the $\Delta G/G_{0}\left(\Delta P\right)$ curves of a channel of height *h* = 2.4 nm for a range of concentrations. We fit the experimental data for all concentrations and confinements with the 1D EDL-advection model. Consistently with previous fits at *c*_0_ = 1 M, we fix the slip length to 20 nm, assuming here that it is independent of the salt concentration $c_{0}$ as a first approximation. Let us mention here that direct measurements of the slip length, with either nanoscale permeability (*27*, *28*) or colloidal probe atomic force microscopy (AFM) (*26*, *29*) measurements, are highly challenging and are still missing in 2D channels to our knowledge. New experiments are needed in particular to fully elucidate the dependence on confinement and salinity. We further assume a concentration regulation of the channel’s surface charge $\Sigma_{\text{ch}}∼c_{0}^{1/3}$ (with $\Sigma_{\text{ch}}=-25\;\text{mC}/m^{2}$ at 1 M) as a one-third exponent was previously reported for the inner walls of carbon nanotubes, which resemble pristine graphite (*25*). The only remaining free parameter is again the surface charge on the activated patch $\Sigma_{\text{inlet}}$.

Overall, this single parameter model fits well the experimental curves across the entire range of concentrations and channel heights (see Fig. 3A and fig. S9). In Fig. 3B, we plot the fitted $\Sigma_{\text{inlet}}$ as a function of salt concentration for all channels and find it to be remarkably independent of the channel’s height. This highly constrained fit of the full dataset confirms the proper description of the mechanosensitivity measurements by the 1D EDL-advection model.

Although independent of *h*, $\Sigma_{\text{inlet}}$ is found to depend on salt concentration as a power law $\Sigma_{\text{inlet}}\propto c_{0}^{\alpha }$, and α=0.71±0.05 here (red dashed line). The regulation exponent we report here is higher than previously measured on carbon nanotubes (*25*) and other carbon surfaces (*14*). Theoretical predictions of charge regulation predict a scaling behavior of the surface charge $\Sigma_{\text{inlet}}\propto c_{0}^{\alpha }$, with an exponent that can vary in the range α∈[1/3,1] depending on conditions (*30*).

In note S2J, we propose a charge-regulation model extending on the description in (*25*) and accounting for a possible salt adsorption on the activated surface. This model predicts a $\Sigma_{\text{inlet}}\propto c_{0}^{2/3}$ scaling, close to our observations α=0.71±0.05. This high value of α nevertheless still contrasts with previous studies (*14*, *25*). We leave further investigations of these effects for future analysis.

### Concentration dependence in experiments versus constrained EDL-advection model

**Fig. 4.**
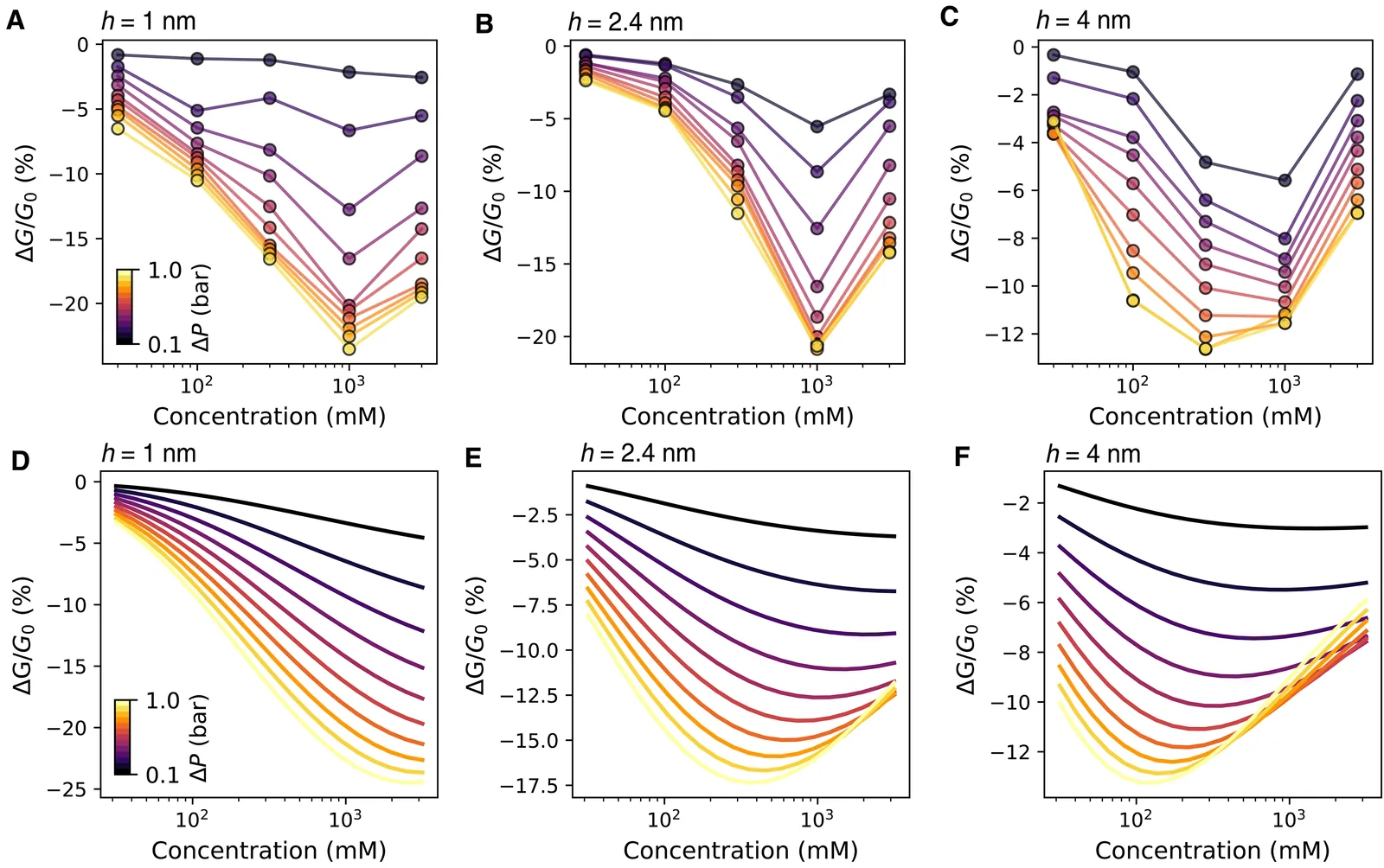
Relative mechanosensitive response as a function of salt concentration for a range of pressure drops in channels of different heights. (**A** to **C**) Concentration-dependent mechanosensitivity curves for channels of different height h∈{1,2.4,4} nm for a set of pressures ranging between 0 and 1 bar. The same dataset is replotted in fig. S9 as a function of pressure instead of concentration. (**D** to **F**) Relative mechanosensitivity $\Delta G/G_{0}\left(c_{0},h,\Delta P\right)$ plotted according to the constrained 1D EDL-advection model without any free parameter. We take *b* = 20 nm, *L* = 5 μm, $\Sigma_{\text{inlet}}=-200\;\text{mC}/m^{2}\times {\left(c_{0}/1M\right)}^{0.7}$, and $\Sigma_{\text{ch}}=-25\;\text{mC}/m^{2}\times {\left(c_{0}/1M\right)}^{0.3}$.

Now that all model parameters have been fully constrained, we plot the experimental measurements of $\Delta G/G_{0}$ against $c_{0}$ (in the range 30 mM to 3 M) for a range of pressure drops and channel heights h∈{1,2.4,4} nm on Fig. 4 (A to C) (data for the h=7 nm channel can be found in fig. S9). For all channels, we find a nonmonotonic trend with concentration: In absolute value, the mechanosensitivity vanishes at both low and high concentration and has a maximum that shifts downward with increasing channel height. In Fig. 4 (D to F), we then report the predicted results of the constrained model—without additional parameters—[with b=20 nm, L=5 μm, $\Sigma_{\text{ch}}=-25\;\text{mC}/m^{2}\times {\left(c_{0}/1M\right)}^{0.3}$, and $\Sigma_{\text{inlet}}=-200\;\text{mC}/m^{2}\times {\left(c_{0}/1M\right)}^{0.7}$]. The model reproduces all the main features of the experimental data: the nonmonotonic behavior with concentration, the downward shift of the maximum with increasing slit height and the quantitative values for $\Delta G/G_{0}$ across parameters. Overall, the quantitative and qualitative agreement between the experimental results and the EDL-advection model confirms the accuracy of our modeling. More broadly, it illustrates how engineering surface charge distributions allows designing an ionic, strain-free, nanofluidic pressure sensor.

In this work, we introduce a new class of mechanosensitive devices based on ion transport in asymmetrically charged nanochannels. Designing a surface charge pattern by exposing one graphite inlet to an oxygen plasma causes a modulation of ionic conductance under pressure drops. In contrast to most existing mechanisms, this effect reports the sign of the pressure drop and does not rely on any structural deformation while maintaining millibar-range sensitivity, comparable to that of biological porins. Our theoretical model explains remarkably well the conductance-pressure response over a wide range of salt concentrations, including the strong enhancement in mechanosensitivity as the channel height drops to 1 nm. Our work opens up new avenues for the design of advanced nanofluidic sensing in solid-state devices based on the precise spatial arrangement of surface charges for specific functions, as demonstrated in biological porins.

## MATERIALS AND METHODS

### Device fabrication

The nanochannel fabrication procedure was refined on the basis of previous studies, and a flow chart is presented in fig. S1. First, we prepared the SiN membrane substrate. The commercially available SiN/Si wafer consists of two SiN layers on both sides of a 500-μm-thick silicon (Si) substrate. A square window was patterned on one side of the SiN layer by photolithography, followed by the removal of the SiN layer via RIE. The wafer was then immersed in a hot KOH solution (70°C) for 12 hours to etch through the middle Si and obtain a freestanding SiN membrane. After that, a rectangular slit was patterned and etched on the other side repeating the photolithography and RIE processes. We then built the nanochannels on the SiN substrate. All graphite and graphene flakes used in this study were prepared using the mechanical exfoliation method on an SiO_2_ substrate. First, a graphite crystal was transferred onto the SiN substrate using the wet transfer method, serving as the bottom layer. After this, RIE was used from the backside of the SiN membrane to open the entrance to the channels. In parallel, we fabricated the spacer layer, which determines the dimensions of the channel. The spacer layer was patterned into tens of parallel strips using a combined EBL and RIE, with a width of ∼150 nm and separated by a similar distance. The cleanliness and height of the spacer layer were confirmed by AFM, which corresponded to the height of the channel. Another graphite crystal was transferred onto the spacer via the wet transfer method, serving as the top layer. This two-layer assembly consisting of the top and spacer layers was lastly transferred onto the bottom crystal on the SiN substrate, ensuring that the channels were aligned perpendicular to the long edge of the rectangular slit. After each transfer, the sample underwent a thorough cleaning with acetone and isopropanol, followed by annealing in an Ar/H_2_ atmosphere at 400°C for 3 hours to remove any potential contamination and polymer residue. Last, a gold strip (3/50-nm Cr/Au) was deposited on top using lithography and e-beam evaporation to serve as a mask for RIE etching, which defines both the number and length of the channels. Before ionic transport measurements, the “activated inlet” nanochannel devices were treated with mild oxygen plasma (O_2_/Ar with Moorfield Nanoetcher) for 1 min. The O_2_/Ar plasma treatment was performed using the nanoETCH Plasma Etching System from Moorfield Nanotechnology following a two-step recipe: (step 1) RF power of 12 W, duration of 3 s, Ar of 16 standard cubic centimeter per minute (SCCM), O_2_ of 8 SCCM, and pressure of 2.6 × 10^−2^ mbar; (step 2) RF power of 8 W and duration of 60 s, with the same gas flows and pressure as in step 1. Pristine-inlet nanochannel devices, on the other hand, were not plasma treated. All fabrication processes were carried out in the clean room.

### Ion transport measurements

For ion transport measurements, the channel device was used to separate two Teflon reservoirs filled with solutions of varying concentrations. The SiN/Si wafer with device under measurement was inserted between two half-cells and sealed with an O-ring to ensure that the nanochannels were the only paths between two half-cells. Two homemade Ag/AgCl electrodes were used as working electrodes to conduct ionic current. To prepare the Ag/AgCl electrode, a current of 1 mA was applied to a Ag wire (0.8 mm in diameter) for 30 min with a voltage range of −1 to −2 V. The prepared Ag/AgCl electrode (dark brown) was then rinsed with deionized water and stored in the 1 M KCl solution. The potential difference between two Ag/AgCl electrodes in a 1 M KCl solution was measured to be less than 5 mV before they could be used for ionic conductance measurements in a range of electrolyte concentrations. KCl solutions of various concentrations were used as the electrolyte. Measurements on the same device were conducted from low to high concentration. Between measurements, the cell was washed thoroughly with the solution of the new concentration before measuring. *I*-*V* curves were recorded using a Keithley 2636B SourceMeter controlled via custom in-house software. When all tests with different concentrations were completed, the cell and tubes were thoroughly washed with deionized water to remove any residues before moving to the next samples. Pressure-driven streaming current measurements were performed using a precision pressure controller (Fluigent), connected to the fluidic cell and operated via OxyGEN software. A constant bias voltage was applied across the nanochannel using a Keithley 2636B SourceMeter, while pressure steps were controlled through the software interface. Chronoamperometry was used to record the ionic current [*I*(*t*)] response at each pressure step. The streaming current at each condition was extracted by averaging the steady-state current values, and the SD was used to quantify measurement uncertainty.

## Supplementary Materials

### This PDF file includes:

Supplementary Notes S1 to S3 Figs. S1 to S17
