# Supplementary material for: Architecting mechanosensitive nanofluidic transport in graphite nanoslits
Source: Sci Adv. 2026 Jul 29;12(31):eaed4451. doi: 10.1126/sciadv.aed4451 (PMC13418753; doi:10.1126/sciadv.aed4451)
Supplement: Supplementary file 1 [file sciadv.aed4451_sm.pdf]

Science Advances
AAAS

Supplementary Materials for

Architecting mechanosensitive nanofluidic transport in graphite nanoslits

Mathieu Lizée *et al.*

Corresponding author: Mathieu Lizée, lizee@fhi-berlin.mpg.de; Qian Yang, qian.yang@manchester.ac.uk; Lydéric Bocquet, lyderic.bocquet@ens.fr

Sci. Adv. **12**, eaed4451 (2026)
DOI: 10.1126/sciadv.aed4451

This PDF file includes:

Supplementary Notes S1 to S3
Figs. S1 to S17

I. SUPPLEMENTARY NOTE S1: ADDITIONAL RESULTS

A. Flowcharts of the fabrication process

On Supplementary Figure S1, we present a detailed flowchart of the fabrication process, along with optical and AFM images of the intermediate steps. The fabrication process consists of two main steps: (1) preparation of the SiN substrate and assembly of the graphite layers, and (2) definition of the channel length and opening of the outlet via metal masking and etching. The optical and AFM images confirm the successful patterning of the spacer layer and the proper assembly of the top layer, which are crucial for achieving the desired channel dimensions and functionality.

B. Concentration-Dependent Conductance and Ion Selectivity in Nanochannels

Prior to pressure-controlled measurements, we conducted a series of concentration-dependent conductance and drift-diffusion experiments to verify the ionic transport characteristics of the fabricated nanochannels. As shown in Supplementary Figure S2**a**, the conductance exhibits a typical surface-charge-governed dependence on KCl concentration, with a clear deviation from bulk behavior in the low-concentration regime. The data are well described by a variable surface charge model, indicating that surface charge plays a dominant role in governing ion transport (15). In addition, the drift-diffusion measurements under asymmetric electrolyte conditions ($c_{\max}/c_{\min} = 10$) reveal cation-selective transport, attributed to the negatively charged surface of graphite. These features – surface-charge-governed conductance and ionic selectivity – are a hallmark signature of confined transport through angstrom-scale slits, verifying both the structural integrity and functional performance of the fabricated nanochannels.

C. Pressure-controlled Ionic Transport in Nanochannels

To probe pressure-driven transport, we applied both positive and negative pressure gradients across a nanochannel device filled with 1 M KCl solution. As shown in Supplementary Figure S3a, the ionic current of the 4 nm channel exhibits a stepwise response to discrete pressure changes under a fixed voltage bias ($\Delta V = 0.1V$). A clear increase (decrease) in

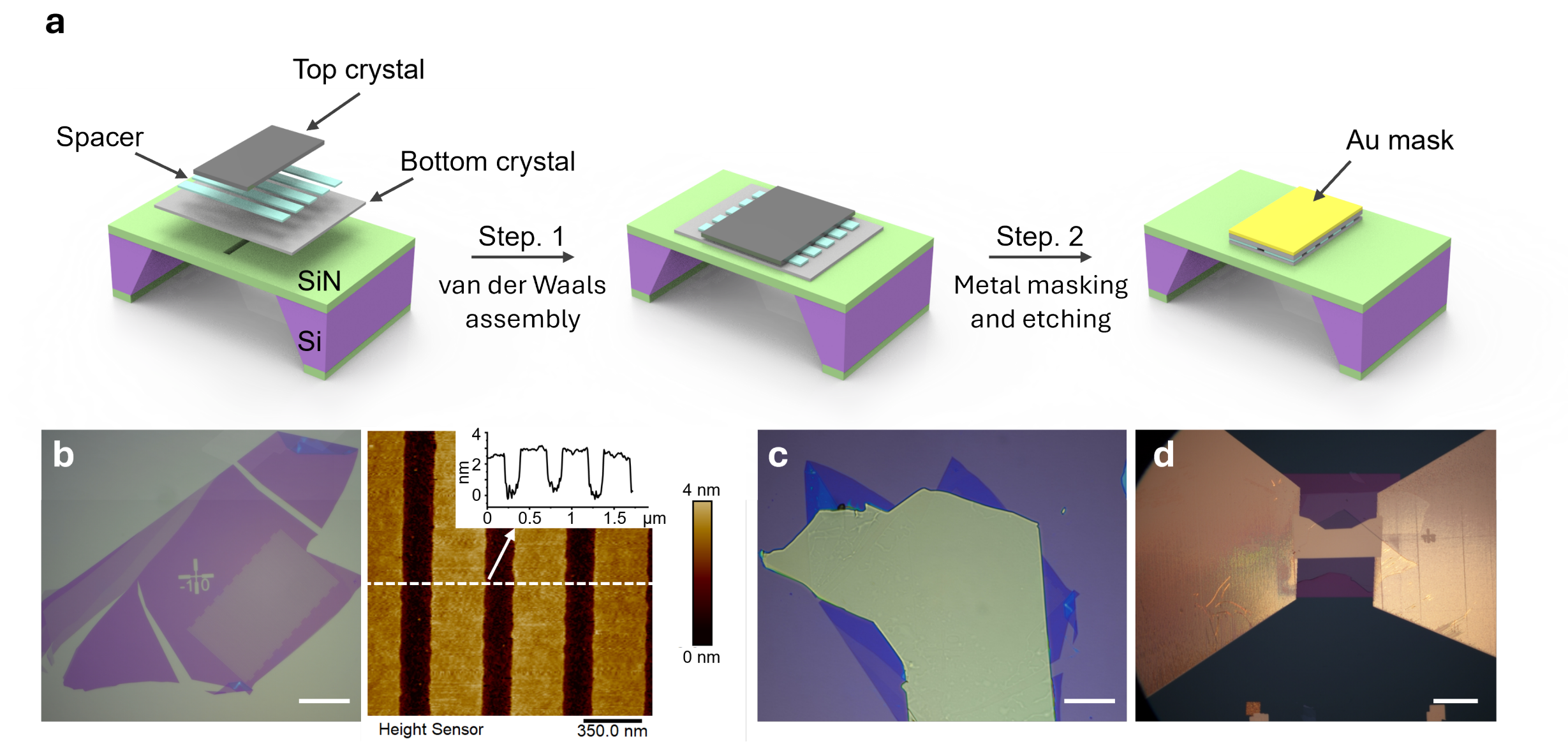

Supplementary Figure S1: **Fabrication and characterization of the nanochannel device.** (**a**)Schematic illustration of the nanochannel fabrication process. In Step 1, three individual crystals are prepared and sequentially assembled on a SiN substrate via van der Waals forces. In Step 2, the nanochannel length is defined and the outlet is opened using metal masking followed by etching. (**b**) Optical image of the spacer crystal after EBL patterning and plasma etching. The right panel shows the corresponding AFM results. Then the spacer crystal was covered with the top crystal (**c**). (**d**) Optical image of the final device with the Au mask. Scale bars: 20 µm in (b) and (c); 50 µm in (d).

current was observed for negative (positive) pressure drops, consistent with 1 nm channel behavior shown in Figure 1 of the main text. Further analysis of the absolute current as a function of pressure drop under various voltage bias ($\Delta V = 0.1V, 0V$) is presented in Supplementary Figure S3**b**. Under zero voltage bias ($\Delta V = 0V$), the observed current arises purely from streaming (advection of the double layer by the flow), displaying a positive linear dependence on pressure, in agreement with previous measurements (7). Since streaming currents are always aligned with the direction of the pressure gradient, a purely streaming-driven response under voltage bias would result in opposite current trends for ($\Delta V \neq 0V$). However, our data show that the current decreases with increasing pressure for both voltage polarities. Moreover, the magnitude of the streaming current is on the order of several tenths of pA, an order of magnitude smaller than the current modulation observed under applied

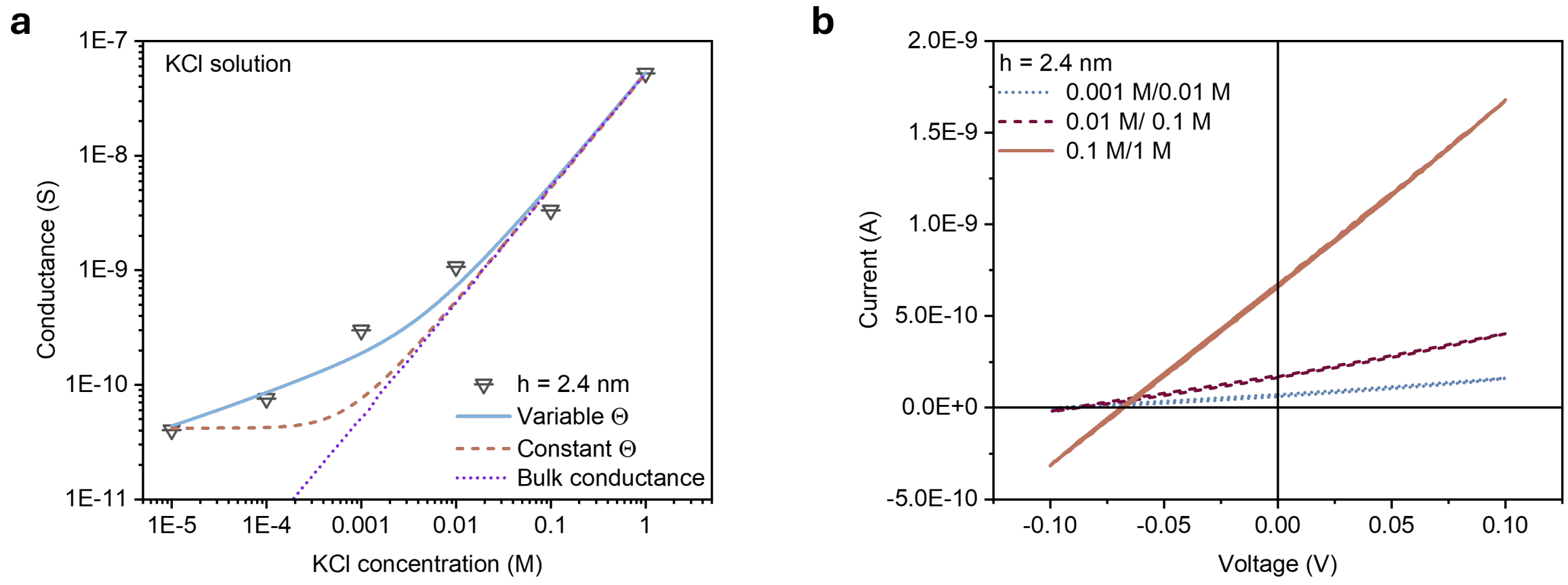

Supplementary Figure S2: **Concentration-dependent conductance and ion selectivity in nanochannels.** (a) Conductance of a 2.4 nm nanochannel measured at different KCl concentrations. The purple dashed curve represents a fit using a constant surface charge model, while the blue solid curves correspond to fits using a variable surface charge model. (b) Drift-diffusion measurements under a fixed concentration gradient ($\Delta = 10$), with varying KCl concentrations.

voltage bias. These results indicate that the observed pressure-induced current changes are not dominated by streaming currents, but rather arise from pressure-dependent changes in nanochannel conductance.

D. Irrelevance of elastic deformations

We naturally turn to elastic deformations of the channel as the driving force of the mechanosensitive conductance. As discussed in the main text, the top layer sagging phenomenon that has been observed in several works (18, 19) is not relevant here. We then consider the silicon nitride membrane, which is known to deform under pressure gradients (3). Our membrane is rather thick (500 nm) but its width is much larger (50 μm) and a large rectangular trench, orthogonal to channels, is opened in the middle to allow the liquid to pass through the membrane. To rule out the membrane's deformation as an origin of mechanosensitivity, a 2.4 nm nanochannel was fabricated on a 100 nm SiN membrane. As shown in Supplementary Figure S5, the 2.4 nm nanochannel with a 100 nm SiN substrate exhibits nearly the same pressure susceptibility of conductance as the channel with a 500 nm SiN substrate (Figure 3 **a** of the main text) despite the 125-drop in stiffness. These results

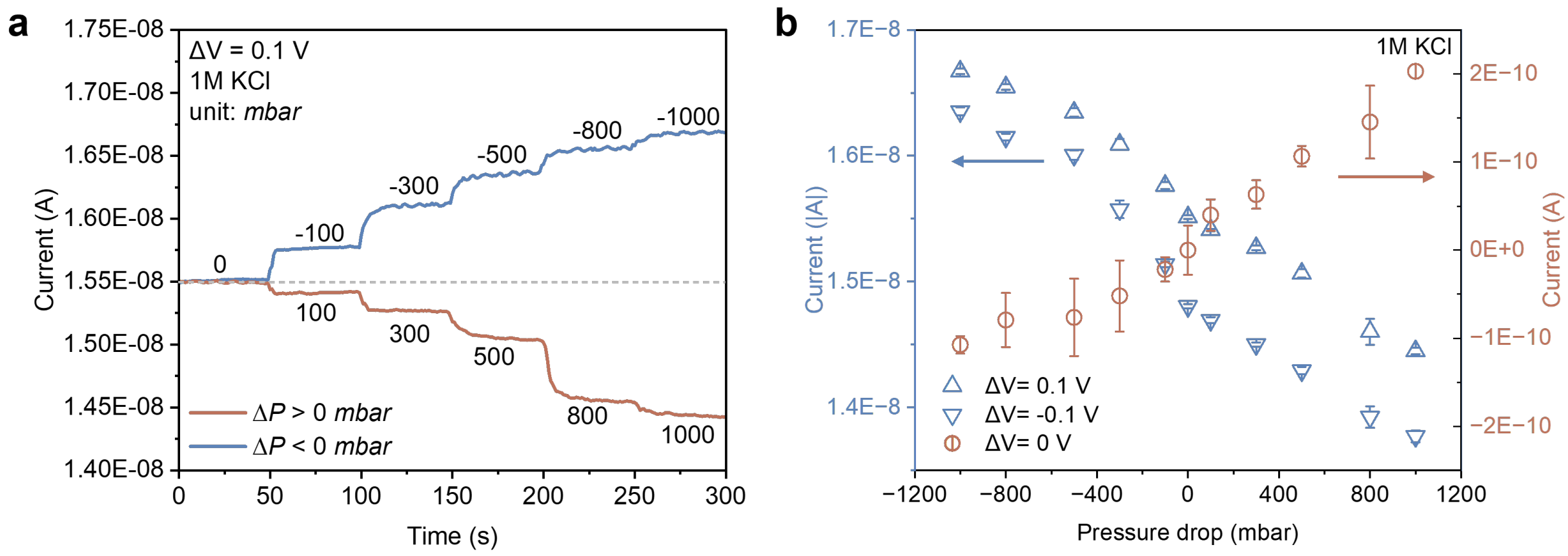

Supplementary Figure S3: **Pressure-controlled ionic transport in nanochannels.** (a) Time series of the current under positive and negative pressure drop steps, for a 4 nm channel in a 1 M KCl solution under a 100 mV voltage bias. (b) Absolute current as a function of pressure drop, under both positive and negative voltage biases.

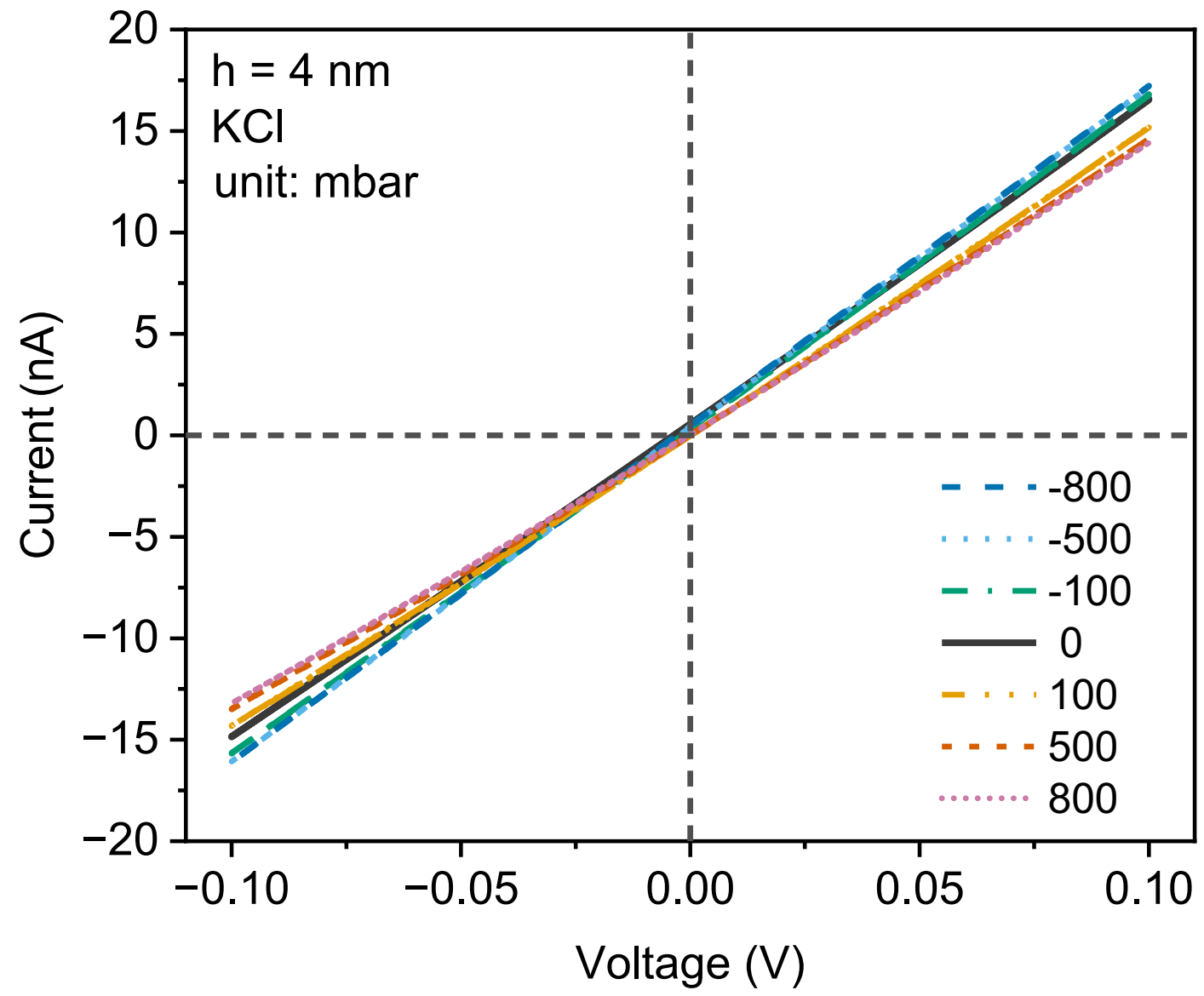

Supplementary Figure S4: **IV curves at various applied pressure** for a 4nm confined system at a KCl concentration of 1 M. Pressure drops are given in mbar. Note the perfect linearity of IV curves under all pressure drops, and the sub-nA streaming currents (at $\Delta V = 0$).

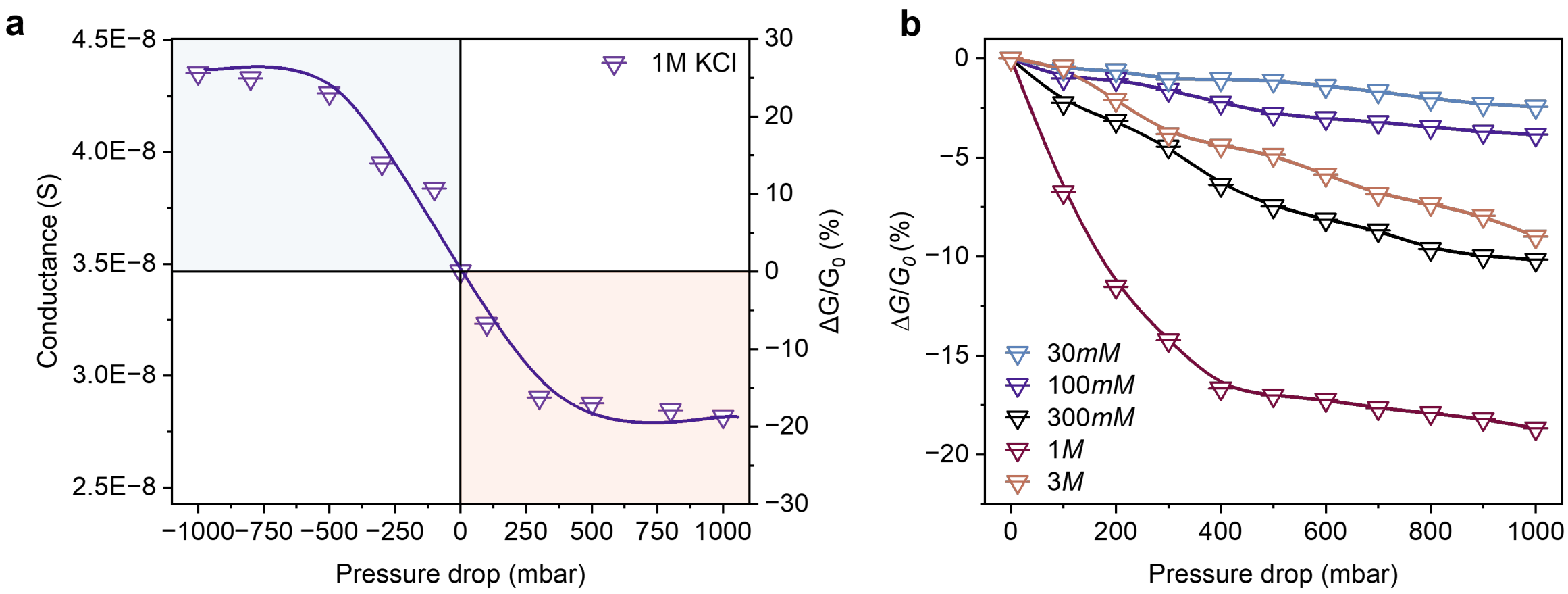

Supplementary Figure S5: **Control measurements on a 100 nm-thick SiN membrane** instead of 500 nm for all other systems. (a) Relative conductance change as a function of pressure drop for a 2.4 nm activated-inlet channel with a 1 M KCl solution. (b) Relative conductance modulation under various KCl concentrations with a positive pressure drop. The negligible difference between the 100 nm and 500 nm SiN membranes indicates that elastic deformation of the SiN membrane under pressure drops is not the dominant factor behind the mechanosensitive conductance.

indicate that the effect of SiN membrane deformation on the mechanosensitivity is negligible.

E. Streaming current measurements

On Supplementary Figure S6, we report streaming current measurements ($\Delta V = 0$, non-zero ΔP) on the activated and non-activated channels. We report no clear increase in the electro-osmotic mobility due to the inlet activation. In a previous study by some of us, we prepared nanochannels with plasma activation across the full channel length (14). In this latter case, the streaming current was very strongly enhanced compared to the pristine case. This observation confirms that only the channel's inlet is activated in our samples. This result also justifies our modeling approach where the current remains proportional to the voltage drop.

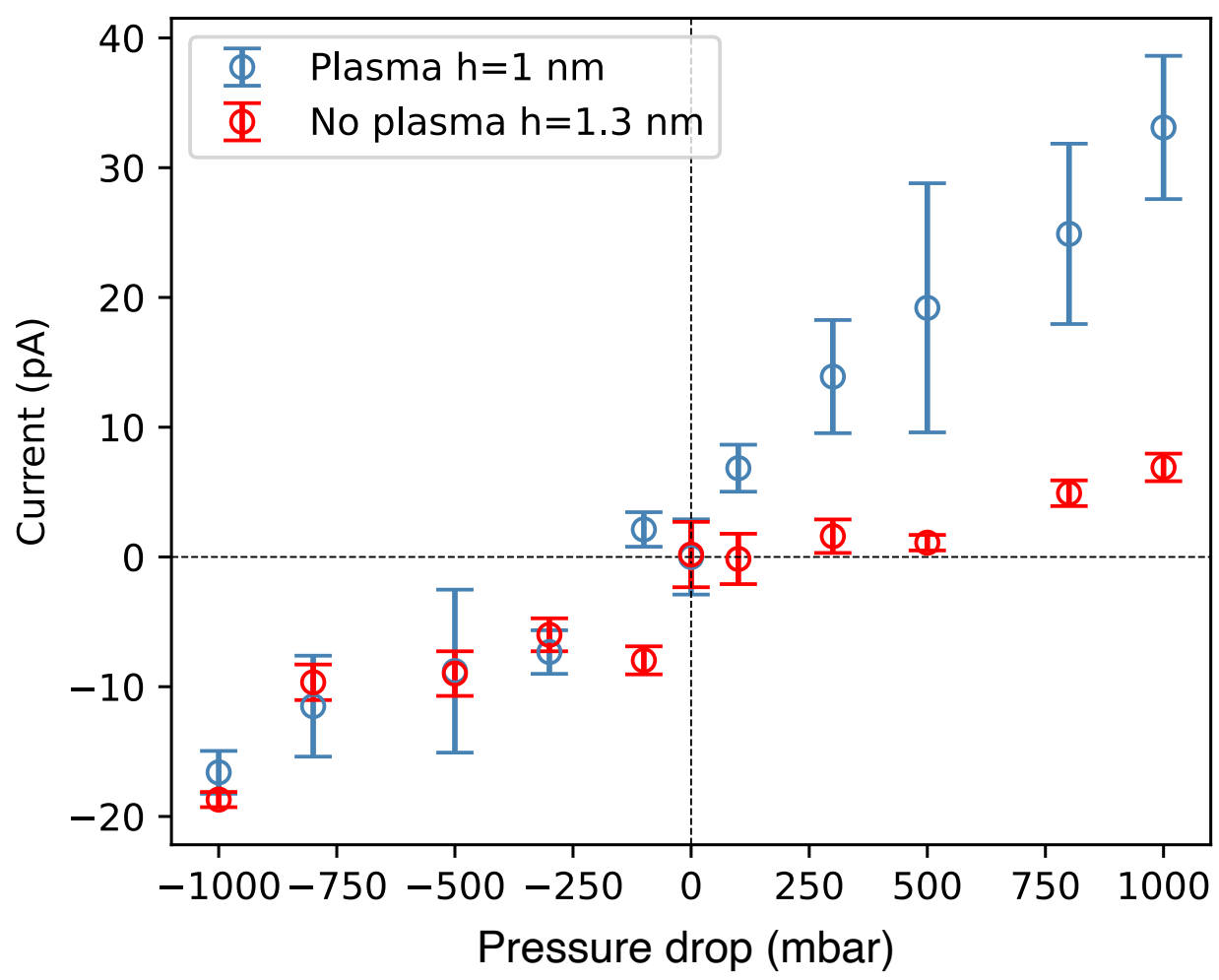

Supplementary Figure S6: **Streaming current measurements** (at zero voltage bias) for the activated ($h = 1$ nm) and non-activated ($h = 1.3$ nm) channels in 1 M KCl solution. The streaming current is not significantly affected by the plasma activation of the inlet, confirming that only the inlet is activated in our samples.

F. Imposed concentration drops across pristine-inlet channels

On Supplementary Figure S7, we report pressure-dependent conductance measurements on *pristine-inlet* channels under concentration gradient. We have shown in the main text (Figure 1**d**) that the conductance modulation in these channels at fixed concentration is very small (under 2% at 1 bar) in 1 M KCl solutions. Here on the other hand, we force a concentration gradient by placing two different solutions in the reservoirs. We find that this imposed concentration drop yields a very similar mechano-conductance $\Delta G(P)/G_0$ to what we measured on the plasma activated inlet channels without the concentration drop. This strongly supports our 2-step modeling process in which we (1) compute the concentration profile due to advection in the presence of the charged inlet and then (2) compute conductance from the concentration profile with the local Ohm's law. We further verify the validity of this model with Comsol simulations in Supplementary Note 3 and Supplementary Figure S17.

We now fit the $\Delta C - \Delta P$ measurements with the simplest version of our model – a simple advection-diffusion competition – and still assuming $b = 20$ nm like we did throughout the main text. The concentration profile in the channel is given by the one-dimensional steady-

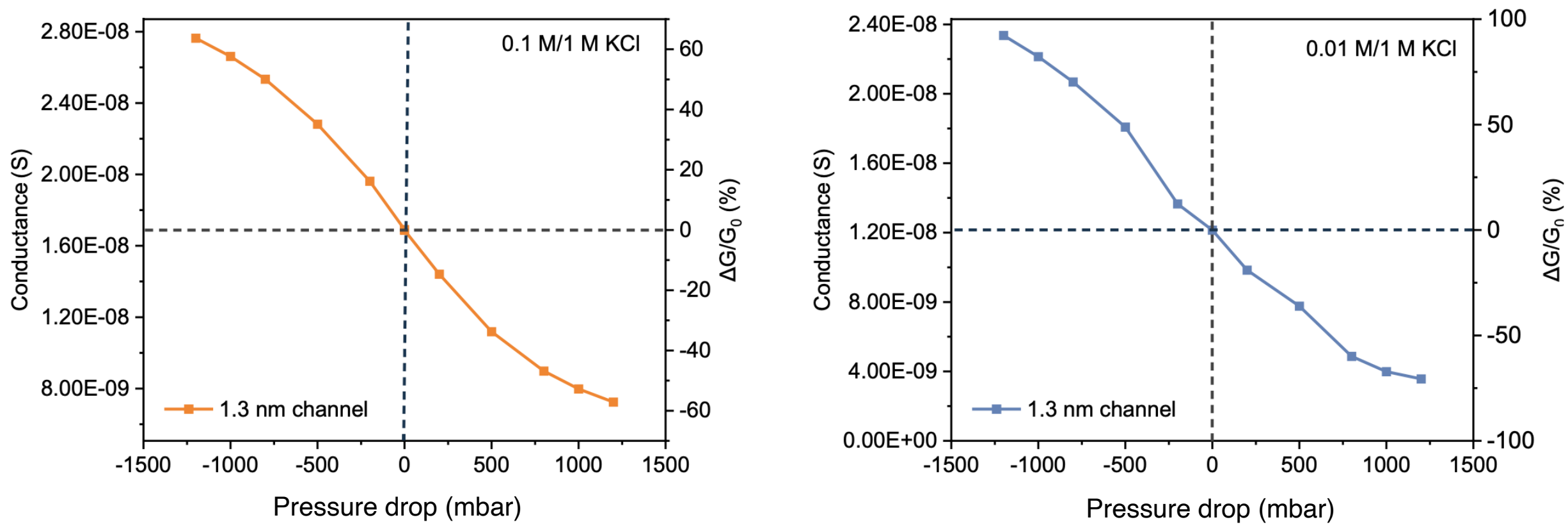

Supplementary Figure S7: **Mechanoconductance measurements under a imposed pressure drop** for the pristine-inlet 1.3 nm channel. The curves reported on panels **a** and **b** have respective concentration drops of 0.1 M/1 M and 0.01 M/1 M. Pressure-dependent conductance measurements without a imposed concentration drop are shown for comparison with Figure 3**c** of the main text and are found to be considerably smaller, of the order of 2% at 1 bar.

state advection-diffusion equation Eq.S1:

$$c(x) - c_0 = \Delta C \cdot \frac{\exp(vx/D) - 1}{\exp(vL/D) - 1} \tag{S1}$$

From the profile, the concentration drop across the channel is given by the local Ohm's law (here we neglect the channel's surface charge and the electro-osmotic flow for simplicity), according to $G/G_0 = \left[\int_0^L dx\, c_0/c_(x)\right]^{-1}$. As shown on Supplementary Figure S8, we reproduce the results without any adjustable parameter. This result further backs up the slip length value of 20 nm used in the main text. It also strongly supports our modeling framework where changes in the pressure-dependent conductance arise from the concentration gradient along the channel, resulting from the competition between advection and diffusion in the presence of a charged inlet.

G. Fitting the mechanosensitivity curves for all concentrations and pressures

On Supplementary Figure S9, we report the fits of all mechanosensitivity curves for all concentrations and channel heights. We find that all data can be fitted with a single slip length of 20 nm, a channel surface charge $\Sigma_{\text{ch}} = -25$ mC/m^2 at 1 M and scaling as $c_0^{1/3}$.

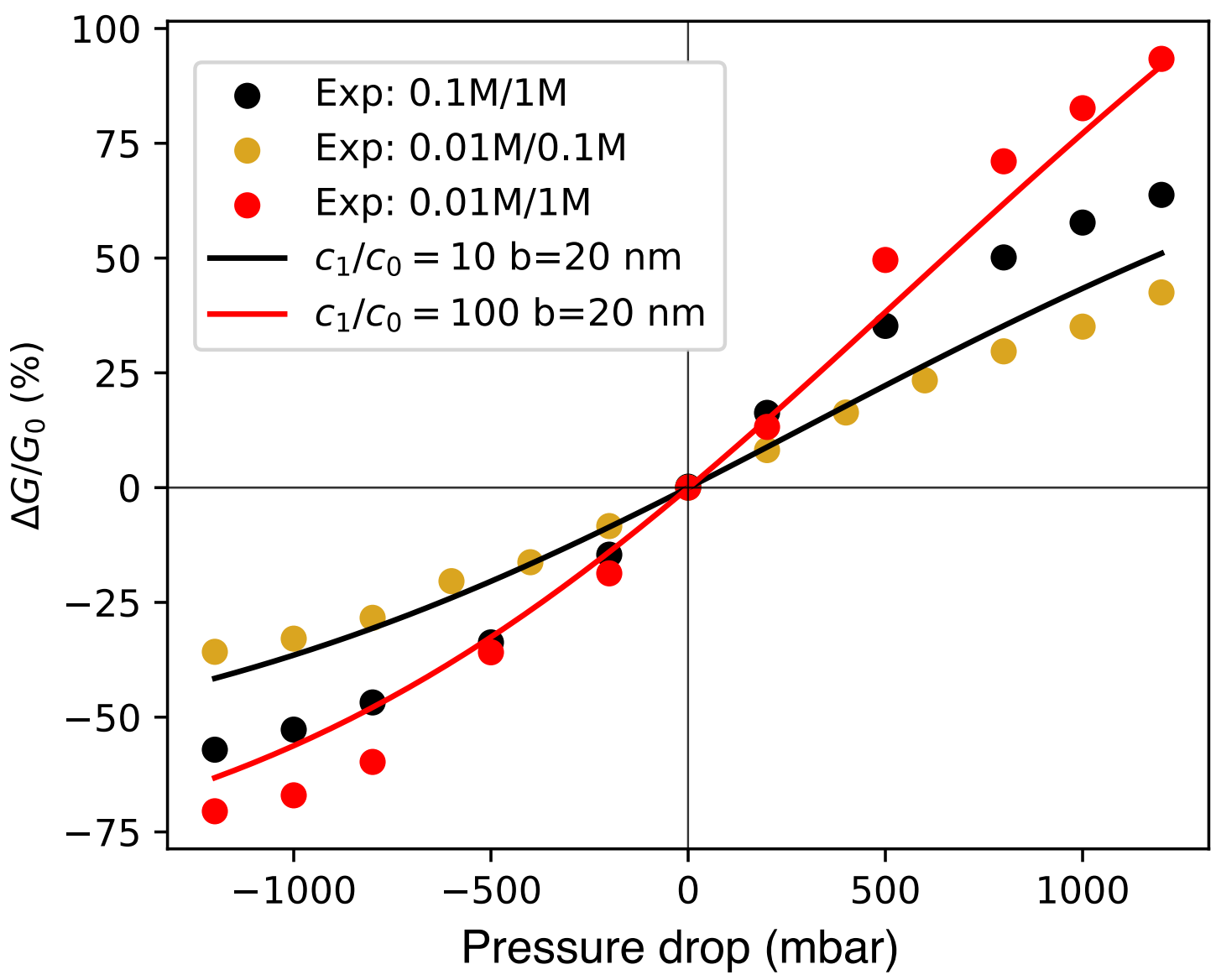

Supplementary Figure S8: **Mechano-conductance measurements under pressure drop** in a 1.3 nm high channel with pristine inlet. The imposed concentration drops are respectively 0.1 M/1 M, 0.01 M/0.1 M and 0.01 M/1 M. Solid lines are the prediction of the advection-diffusion model for the relative mechanoconductance, with a slip length of 20 nm. Pressure-dependent conductance measurements without a imposed concentration drop are shown for comparison with Figure 3**c** of the main text.

The inlet surface charge obtained from these fits is reported in Figure 4**b** of the main text.

II. SUPPLEMENTARY NOTE S2: THEORETICAL DETAILS ON THE SURFACE-CHARGE ADVECTION MODEL

A. Flow velocity in the channel

We consider that the hydraulic resistance is dominated by the channel. Thus, the average velocity of water in the channel from a pressure drop ΔP is given by:

$$v_0 = \frac{h^2}{12\eta L}\Delta P \left(1 + 6\frac{b}{h}\right) \tag{S2}$$

where h is the channel height, L is the channel length, b the slip length, and η the fluid dynamic viscosity. This velocity sets the Péclet number:

$$\mathrm{Pe} = \frac{v_0 L}{D} = \frac{h^2 \Delta P}{12\eta D}\left(1 + 6\frac{b}{h}\right) \tag{S3}$$

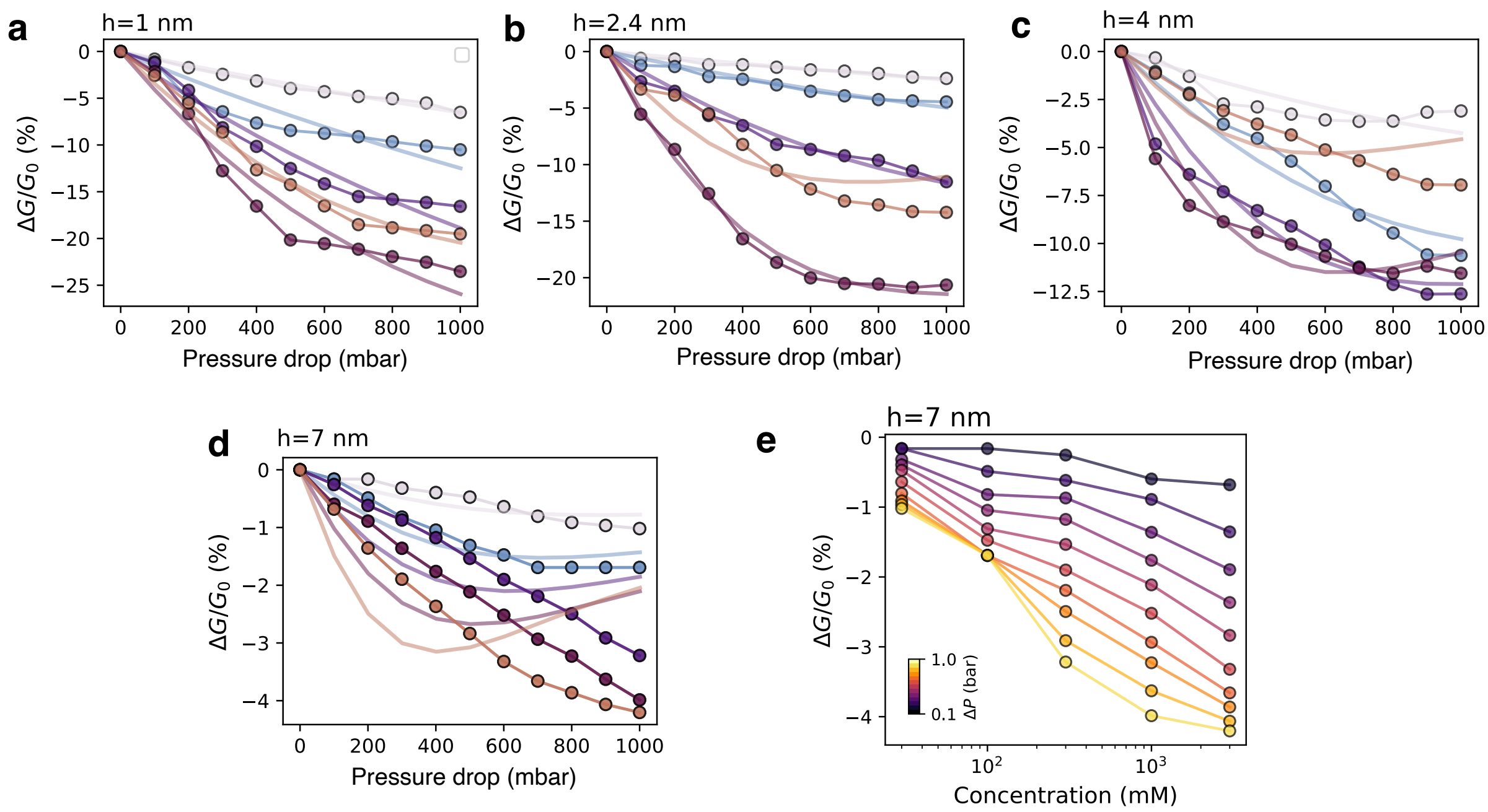

Supplementary Figure S9: **Dependence on concentration of the mechanosensitive effect and predictions of the theoretical model** (**a-d**) Mechano-conductance *versus* pressure curves for 4 systems with height $h \in \{1, 2.4, 4, 7\}$ nm for ΔP ranging between 0 and 1 bar. The raw data are taken from Figure 4**a-c** of the main text. Solid lines are fit with the theoretical model, assuming a slip length of 20 nm, a pristine graphite surface charge of -25 mC/m^2 at c_0=1 M and a regulation of $\Sigma_{\text{ch}} \sim c_0^{1/3}$ consistent with existing measurements on carbon nanotubes (25). The inlet surface charge obtained from these fits is reported in Figure 3**b** of the main text. **(e)** Concentration-dependent response plotted for various pressure-drops for the 7 nm thick channel.

which controls the strength of polarization and conductance variation. In the experiment, $h \ll b$ and we can assume a plug flow:

$$\text{Pe} \approx \frac{hb\Delta P}{2\eta D} \tag{S4}$$

B. Convergent flow at the inlet and equivalent channel geometry

In principle, the geometry of the entrance for fluids to flow into the nanochannels from the reservoirs, as sketched in Figure 2a of the main text, is strongly convergent, similar to

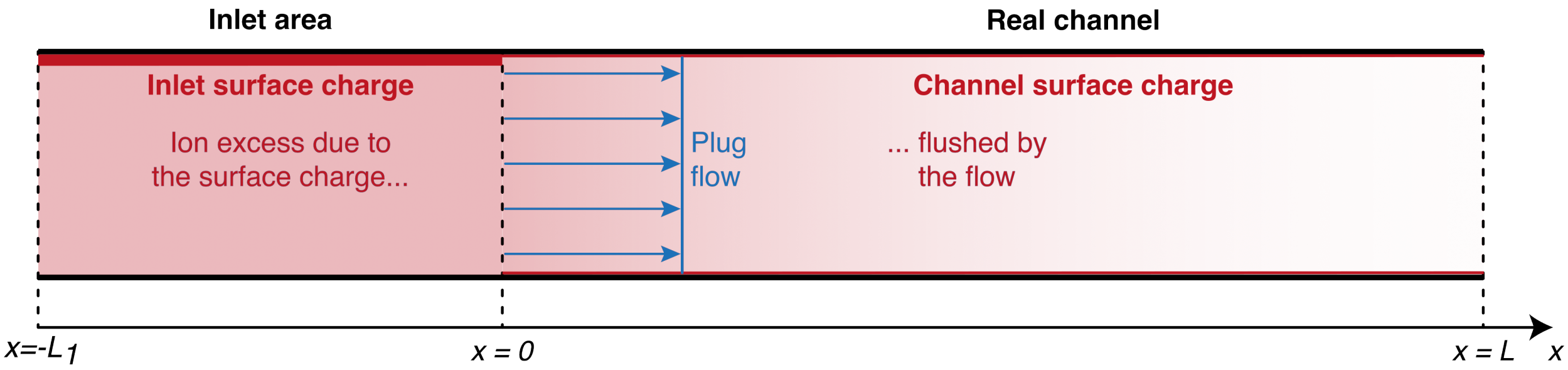

Supplementary Figure S10: Sketch of the model. We model the inlet region as a channel of height h and length L_1 with surface charge Σ_{inlet}.

the Jeffery-Hamel flow, with a 2D profile behaving as

$$\mathbf{v}(\vec{x}, t) = v_0\, h \frac{\sin 2\theta}{r}\, \mathbf{e}_r \tag{S5}$$

where θ is the polar angle of the vector $\vec{x}$ (with $\theta \in [0; \pi/2]$ here). Note that such a flow profile accounts for convergence but neglects slippage.

However, in the present problem, we are mainly interested in the convective transport of ions within the nanometric electric double layer (EDL) close to the activated inlet surface and only the flow within the EDL, with height $\lambda_{\text{D}} \sim \mathcal{O}(1)$ nm, will contribute to the convective transport of the ions. Accordingly, we made a simplifying approximation for the entrance flow by focusing on this thin layer close to the inlet's surface. We restricted the description by considering the inlet region as a channel of height h over a length L_1 see Supplementary Figure S10. This schematic geometry allows us to avoid treating the high velocity gradients at the highly convergent geometry of the real inlet (we solved a more accurate geometry with finite element calculations, see Supplementary Note 3 and the mesh on Supplementary Figure S12).

This simplification reduces the considerable complexity of the electrohydrodynamic problem, while keeping the main physical ingredients. It is furthermore justified *a posteriori* as it allows obtaining analytical predictions for the electrohydrodynamic transport which are in agreement with the experimental findings, as well as with the finite element calculations discussed below.

C. Poisson-Nernst-Planck-Stokes (PNPS) transport

In line with the discussion above, the geometry consists of the 'real' nanochannel of height h for $0 < x < L$, with surface charge Σ_{ch} on both interfaces and slip length $b_{\text{ch}} \gg h$, continued by the inlet region modeled as a channel of height h for $-L_1 < x < 0$, with surface charge Σ_{inlet} on the top interface and slip length $b_{\text{inlet}} \gg h$; see Supplementary Figure S10. We denote $\mathscr{A} = hw$ the channel cross section where w is the channel width and h its height.

The bulk salt concentration is c_0 and we have a concentration profile $c(x)$ of the channels. We define the local and global Dukhin numbers:

$$\text{Du}(x) = \frac{|\Sigma(x)|}{hc(x)}, \qquad \text{Du}_{\text{ch}} = \frac{|\Sigma_{\text{ch}}|}{hc_0}, \qquad \text{Du}_{\text{inlet}} = \frac{|\Sigma_{\text{inlet}}|}{hc_0} \tag{S6}$$

where $\Sigma(x)$ is the surface charge density at position x.

D. Poisson-Nernst-Planck (PNP) transport

In the system under scrutiny here, there is a separation of length scales between the size of the EDL and the characteristic scales over which the geometry or flow varies laterally. The lengthscale over which the surface charge varies due to plasma exposure is given by the channel height h which is larger than the Debye length λ_{D}. This allows us to assume the Poisson-Boltzmann equilibrium in each vertical cross section of the channel. Following the description introduced by Poggioli *et al.* (22) for thin EDL, we can then separate the vertical and horizontal scales and derive one-dimensional transport equations for the total ionic concentration. The total salt current is accordingly obtained as (22):

$$J_0 = -\mathscr{A} D \left[(1 + \mathscr{F}[\text{Du}(x)])\partial_x c - \text{Du}(x) \frac{ec}{k_B T} \partial_x \phi \right] \tag{S7}$$

where the first term accounts for the diffusion with the assumption of equal mobility of the anion and cation (which is very good for KCl) and the second term for electromigration, with ϕ the electric potential. Note that the excess salt concentration due to the surface charge (22) is accounted for in $\mathscr{F}[\text{Du}]$:

$$\mathscr{F}[\text{Du}] = \frac{2\lambda_D}{h} \left\{ \sqrt{\left[\frac{h}{2\lambda_D} \text{Du} \right]^2 + 1} - 1 \right\} \tag{S8}$$

In the high and low Dukhin limits:

$$\begin{aligned}
&\text{(high Du)} \qquad \mathscr{F}(\mathrm{Du}) \simeq \mathrm{Du} \\
&\text{(low Du)} \qquad \mathscr{F}(\mathrm{Du}) \simeq \frac{h}{4\lambda_D}\mathrm{Du}^2
\end{aligned}$$

In general, the continuity equation of the ion current should be completed by the continuity of the electric current. However, in this model we neglect the lateral electric fields since charge separation happens only at the scale of the EDL which is much smaller than any lateral size of the problem and even than h in most experimental conditions. As a consequence, we drop the electromigration term in Eq.(S7) and focus on the diffusive and convective contributions to the ion flux.

E. Advection

Now we add the advection term to the flow, which flushes the salt concentrations in the EDL and above. The corresponding salt current is:

$$J_{\mathrm{conv}}(x) = \mathscr{A}\,\langle c(x,z)v(x,z)\rangle_z \tag{S9}$$

where $\langle\cdot\rangle_z$ denotes the average over the channel height h. Since the flow profile is assumed to be plug-like, we deduce

$$J_{\mathrm{conv}}(x) \approx \mathscr{A}c(x)(1+\mathscr{F}[\mathrm{Du}(x)])v_0 \tag{S10}$$

Summing the diffusive and convective contributions to the salt flux, Eq.(S7)-(S10), one obtains finally

$$J = -\mathscr{A}D(1+\mathscr{F}[\mathrm{Du}(x)])\left(\partial_x c(x) - \mathrm{Pe}\,\frac{c(x)}{L}\right) \tag{S11}$$

F. Concentration profile and effective conductance

For each channel, the concentration obeys the partial differential equation

$$J\times\frac{L}{\mathscr{A}D} = (1+\mathscr{F}[\mathrm{Du}(x)])(-L\partial_x c(x) + \mathrm{Pe}\,c(x)) \tag{S12}$$

where the salt flux J is constant versus x. Boundary conditions are $c(-L_1) = c(L) = c_0$ as well as the continuity of the ion density $c(x)$ at $x = 0$. In the general case, this equation

has to be solved numerically. But an analytical expression can be obtained in the low Péclet regime, where the concentration profile modifications are small.

Finally, the conductance is calculated from the number of mobile charges in the nanochannel, which we assume is limiting. According to Ohm's law and taking into account the contribution of the surface conduction,

$$G = 2hw\frac{e^2 D}{k_\mathrm{B} T}\left[\int_0^L \frac{dx}{c(x) + c_0\,\mathrm{Du_{ch}}}\right]^{-1} \tag{S13}$$

In the absence of flow, $c(x) = c_0$ and

$$G_0 = 2hw\frac{e^2 D}{k_\mathrm{B} T}\frac{c_0}{L}(1 + \mathrm{Du_{ch}}) \tag{S14}$$

The relative conductance then reads:

$$\frac{\Delta G}{G_0} = \frac{1}{1+\mathrm{Du_{ch}}}\left[\int_0^L \frac{\frac{dx}{L}}{\frac{c(x)}{c_0} + \mathrm{Du_{ch}}}\right]^{-1} - 1 \tag{S15}$$

G. Low Péclet regime

In the low Péclet regime the salt concentration changes weakly: $c(x) \approx c_0$. Therefore, we approximate the Dukhin number using the bulk salt concentration:

$$\mathrm{Du}(x) \approx \mathrm{Du_{inlet}} \text{ for } x < 0, \quad \mathrm{Du}(x) \approx \mathrm{Du_{ch}} \text{ for } x > 0 \tag{S16}$$

Thus, the equation for the salt concentration can be solved for each side of the system:

$$c(x) = \frac{JL}{\mathscr{A} D(1 + \mathscr{F}[\mathrm{Du}])\mathrm{Pe}} + \mathscr{C} e^{\mathrm{Pe}\, x/L} \tag{S17}$$

where we have a constant $\mathscr{C}$ to fix for each side of the system.

Using the boundary conditions $c(-L_1) = c(L) = c_0$, we then obtain the following linear profiles in the low Péclet regime:

$$c(x) = c_0\left(1 + \mathrm{Pe}\,\frac{x-L}{L}\right) - \frac{JL}{\mathscr{A} D(1+\mathscr{F}[\mathrm{Du_{ch}}])}\frac{x-L}{L} \qquad \text{for } 0 < x < L \tag{S18}$$

$$= c_0\left(1 + \mathrm{Pe}\,\frac{x+L_1}{L}\right) - \frac{JL}{\mathscr{A} D(1+\mathscr{F}[\mathrm{Du_{inlet}}])}\frac{x+L_1}{L} \qquad \text{for } -L_1 < x < 0 \tag{S19}$$

where we used that J is of the order of Pe and neglected terms of higher order. The continuity at $x = 0$ further requires:

$$c_0\,(1 - \mathrm{Pe}) + \frac{JL}{\mathscr{A} D(1+\mathscr{F}[\mathrm{Du_{ch}}])} = c_0\left(1 + \mathrm{Pe}\,\frac{L_1}{L}\right) - \frac{JL}{\mathscr{A} D(1+\mathscr{F}[\mathrm{Du_{inlet}}])}\frac{L_1}{L} \tag{S20}$$

that is:

$$J = \frac{\mathscr{A} D c_0}{L} \frac{(L+L_1)(1+\mathscr{F}[\mathrm{Du_{ch}}])(1+\mathscr{F}[\mathrm{Du_{inlet}}])}{L(1+\mathscr{F}[\mathrm{Du_{inlet}}]) + L_1(1+\mathscr{F}[\mathrm{Du_{ch}}])} \mathrm{Pe} \tag{S21}$$

and finally:

$$\frac{\Delta c}{c_0} = \frac{c(0)-c_0}{c_0} = \frac{\mathscr{F}[\mathrm{Du_{inlet}}]) - \mathscr{F}[\mathrm{Du_{ch}}]}{1+\mathscr{F}[\mathrm{Du_{ch}}] + \frac{L}{L_1}(1+\mathscr{F}[\mathrm{Du_{inlet}}])} \mathrm{Pe} \tag{S22}$$

This can be written as

$$\frac{\Delta c}{c_0} = \frac{c(0)-c_0}{c_0} = \Gamma \times \mathrm{Pe} \tag{S23}$$

where the coefficient Γ has the expression

$$\Gamma = \frac{\mathscr{F}[\mathrm{Du_{inlet}}]) - \mathscr{F}[\mathrm{Du_{ch}}]}{1+\mathscr{F}[\mathrm{Du_{ch}}] + \frac{L}{L_1}(1+\mathscr{F}[\mathrm{Du_{inlet}}])} \tag{S24}$$

Finally, one deduces the conductance change $\Delta G/G_0 = (G-G_0)/G_0$ as

$$\frac{\Delta G}{G_0} = \frac{1}{2}\frac{1}{1+\mathrm{Du_{ch}}}\frac{\mathscr{F}[\mathrm{Du_{inlet}}]) - \mathscr{F}[\mathrm{Du_{ch}}]}{1+\mathscr{F}[\mathrm{Du_{ch}}] + \frac{L}{L_1}(1+\mathscr{F}[\mathrm{Du_{inlet}}])} \mathrm{Pe} \tag{S25}$$

The length L_1 corresponds to the diffusive lengthscale to relax the salt concentration drop Δc from the inlet region to the far reservoir. Physically, the inlet region cannot sustain both the excess of ions due to Σ_{inlet} and the salt flux J imposed by the nanochannel without the support of a gradient of salt concentration, resulting in the appearance of a lengthscale describing the transition between the inlet region and the far reservoir. It is challenging to provide a quantitative value for L_1 since it takes into account a complex geometry of the inlet region and subtle physical phenomena into a single simplifying and effective parameter. Qualitatively, we expect L_1 to be large compared to all microscopic parameters (h, b, λ_D). As a rough simplification, we then assume that $L_1 = L$ in the following. Note that our qualitative predictions do not depend on this assumption. We have accordingly:

$$\frac{\Delta G}{G_0} = \frac{1}{2}\frac{\mathscr{F}[\mathrm{Du_{inlet}}]) - \mathscr{F}[\mathrm{Du_{ch}}]}{(1+\mathrm{Du_{ch}})(2+\mathscr{F}[\mathrm{Du_{ch}}] + \mathscr{F}[\mathrm{Du_{inlet}}])} \mathrm{Pe} \tag{S26}$$

a. Large Dukhin regime. We have $\mathscr{F}[\mathrm{Du}] \approx \mathrm{Du}$ thus, taking into account the number of surfaces,

$$\frac{\Delta G}{G_0} \approx \frac{1}{2}\frac{\mathrm{Du_{inlet}} - \mathrm{Du_{ch}}}{\mathrm{Du_{ch}}(\mathrm{Du_{inlet}} + \mathrm{Du_{ch}})} \mathrm{Pe} \tag{S27}$$

Note that the relative conductance variation vanishes for $\mathrm{Du} \gg 1$, which is expected for very low concentrations.

b. Low Dukhin regime. In this regime, $\mathscr{F}[\mathrm{Du}] \approx \frac{h}{4\lambda_D}\mathrm{Du}^2$ and

$$\frac{\Delta G}{G_0} = \frac{h}{8\lambda_D}\left(\mathrm{Du}_{\mathrm{inlet}}^2 - \mathrm{Du}_{\mathrm{ch}}^2\right)\mathrm{Pe} \tag{S28}$$

Note that the relative conductance variation also vanishes for $\mathrm{Du} \ll 1$, which is expected for very high concentrations.

H. Flow renormalization by diffusio-osmotic transport

The flush induces an ionic gradient at the boundaries of the nanochannel $\Delta c/c$ and this may induce osmotic driving. This echoes some experimental findings in carbon nanotubes by Lokesh et al. (23).

If the EDL is thin with respect to the channel height, a diffusio-osmotic (DO) flow will be generated under the concentration gradient (24), which will add to the pressure-driven flow. The (plug-like) DO velocity takes the expression

$$v_{\mathrm{DO}} = K_{\mathrm{DO}}\frac{\Delta c}{L} \tag{S29}$$

where $\Delta c = c(0) - c(L)$ and the DO mobility reads (24)

$$K_{\mathrm{DO}} = \frac{k_B T}{\eta}\lambda^2\left(1 + \frac{b}{\lambda}\right) \approx \frac{k_B T}{\eta}\lambda b \tag{S30}$$

where λ is the size of the diffusion layer and b the slip length on the nanochannel surface (we anticipated that $b \gg h$). For salt $\lambda \sim \lambda_D$, but this is expected to saturate to a minimal molecular size for very high salt concentration $\lambda \sim \sigma$, with σ the ion diameter (typically the size of the Stern layer at the boundaries).

Now the concentration drop Δc is proportional to the bare, pressure-driven velocity v_0, according to $\frac{\Delta c}{c_0} = \Gamma\,\frac{L}{D}v_0$, and the DO velocity is proportional to v_0 as

$$v_{\mathrm{DO}} = \frac{k_B T}{\eta}\lambda b \times c_0\,\Gamma \times \frac{v}{D}, \tag{S31}$$

Altogether, the total, effective velocity in the nanochannel reads

$$v_{\mathrm{eff}} = v_0 + v_{\mathrm{DO}} \tag{S32}$$

so that

$$v_{\mathrm{eff}} = \left(1 + c_0\frac{k_B T}{\eta D}\lambda b \times \Gamma\right) \times v_0 \tag{S33}$$

and the effective Péclet number, renormalized by the diffusio-osmotic transport, is calculated as

$$\mathrm{Pe}_{\mathrm{eff}} = \mathrm{Pe}\left(1 + c_0 \frac{k_B T}{\eta D} \lambda b \times \Gamma\right) \tag{S34}$$

I. High Péclet regime

For large Péclet number one needs to take into account nonlinearities involved in the dependence of the local Dukhin number on the concentration $c(x)$. This can be done numerically by solving the equation for the concentration profile, in Eq.(S12), for each channel and imposing the continuity at $x = 0$.

On Supplementary Figure S11, we plot the results of numerically solving the non-linear model, taking into account the diffusio-osmotic correction to the flow. We find a remarkable agreement with both experiments and the Comsol simulations presented below, note for example the asymmetry in the pressure response at high slippage (panel **c**) and compare with Comsol results on Supplementary Figure S15 **b**.

J. Surface charge regulation

We start from (25) and denote c_a the bulk concentration of the species which is adsorbed. The equilibrium of chemical potential reads:

$$\mu_a = k_B T\, \ln\!\left(\Sigma\, \lambda^2\right) - eV_s + U_{\mathrm{ads}} = k_B T\, \ln\!\left(c_a\, \lambda^3\right) \tag{S35}$$

where V_s is the electric potential at the interface, U_{ads} the adsorption potential and λ a microscopic lengthscale. Thus

$$|\Sigma| = c_a \lambda K_A\, e^{\phi_s} \tag{S36}$$

where

$$K_A = e^{-U_{\mathrm{ads}}/k_B T}, \qquad \phi_s = \frac{eV_s}{k_B T} \tag{S37}$$

Following (25) and assuming no Debye overlap, we can use the properties of the Poisson-Boltzmann equation to get:

$$\frac{1}{2}\left(\frac{\Sigma}{c_a \lambda K_A} + \frac{c_a \lambda K_A}{\Sigma}\right) = \frac{2}{\left(\kappa\, \ell_{GC}\right)^2} = \frac{\pi \Sigma^2 \ell_B}{c_0} \tag{S38}$$

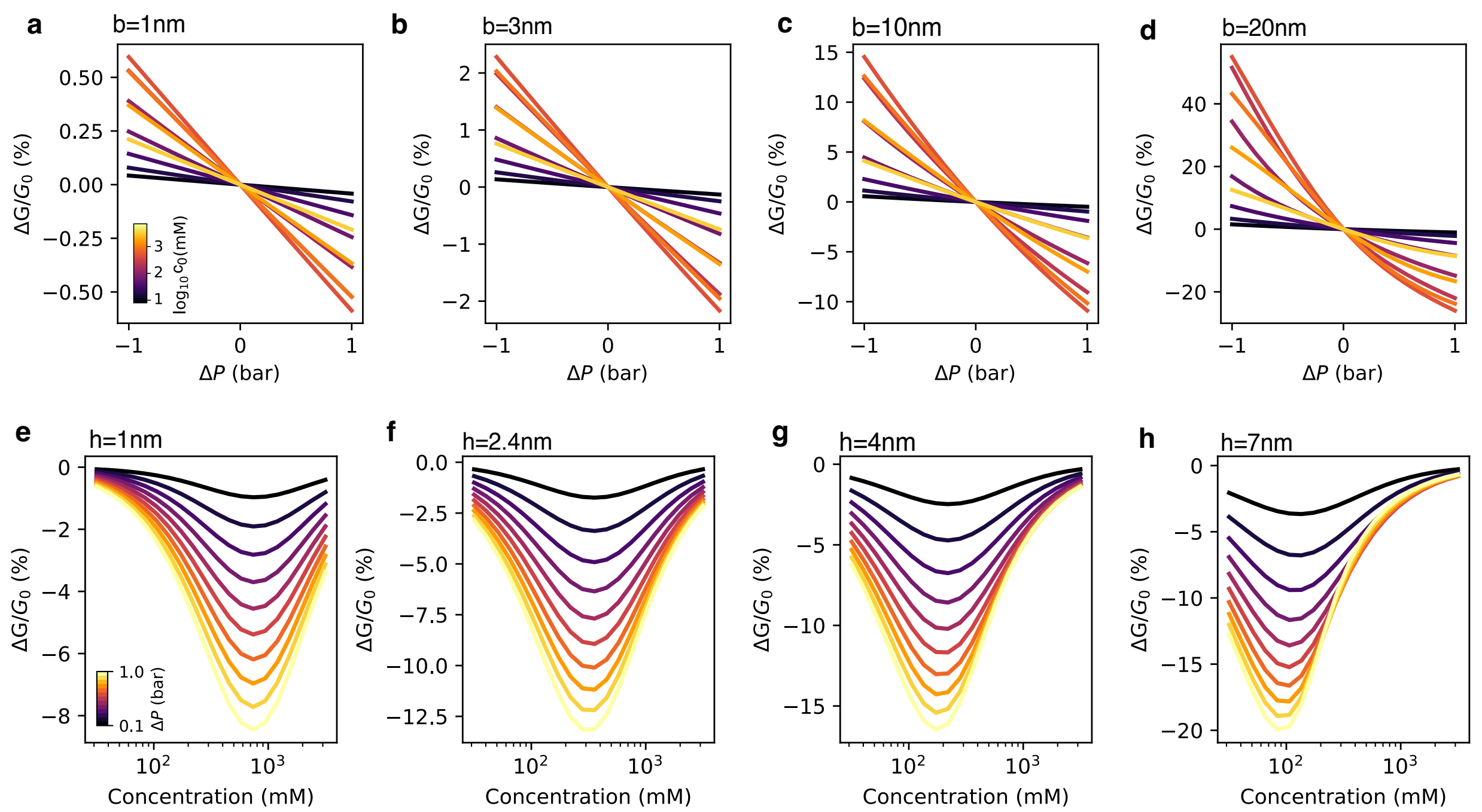

Supplementary Figure S11: Numerical solution with fixed surface charges for $L = 5\ \mu$m, $\Sigma_{\text{inlet}} = 100$ mC/m^2, $\Sigma_{\text{ch}} = 0$ and a range of pressure drops and salt concentrations c_0. **a-d** Solutions for $h = 1$ nm and $b \in \{1, 3, 10, 20\}$ nm. We clearly see the non-monotonic dependence on concentration and the asymmetry in the stronger saturation at positive pressure which we also observe in the Comsol solution. Note that in contrast to the fits in Figure 4 of the main text and Supplementary Figure S9, here Σ_{inlet} is independent of concentration. **e-h** Solutions for $b = 20$ nm and $h \in \{1, 2.4, 4, 7\}$ nm plotted as a function of concentration for fixed pressure drops.

where ℓ_{GC} and ℓ_{B} are the Gouy-Chapman and Bjerrum lengthscales and c_0 is the salt concentration which controls the EDL. c_0 may be the same as c_a, but may also correspond to a different species. This equation simplifies in various regimes of the Poisson-Boltzmann equation.

a. Debye-Hückel regime (low surface charge). We assume $|\Sigma| \ll c_a \lambda K_A$ then:

$$\frac{c_a \lambda K_A}{2\Sigma} = \frac{\pi \Sigma^2 \ell_B}{c_0} \tag{S39}$$

and finally

$$\Sigma = \left(\frac{c_a c_0 \lambda K_A}{2\pi \ell_B} \right)^{1/3} \propto c_a^{1/3} c_0^{1/3} \tag{S40}$$

We find an exponent 1/3 if the adsorbed ions are different from the salt species (*e.g.* OH^- adsorption), while an exponent 2/3 is found if the adsorbed ions belong to the salt species

$(c_a = c_0)$.

b. Gouy-Chapman regime (high surface charge). We assume $|\Sigma| \gg c_a \lambda K_A$, then:

$$\frac{\Sigma}{2c_a\lambda K_A} = \frac{\pi\Sigma^2\ell_B}{c_0} \tag{S41}$$

and finally:

$$\Sigma = \frac{c_0}{2\pi\ell_B c_a\lambda K_A} \propto \frac{c_0}{c_a} \tag{S42}$$

We find an exponent 1 if the adsorbed ions are different from the salt species (*e.g.* OH^- adsorption), and a vanishing exponent is found if the adsorbed ions belong to the salt species.

c. Experimental regime. The channel surface charge is always small and is therefore described by the Debye-Hückel regime. The inlet surface charge is larger, so that $\ell_{\mathrm{GC}} \sim \lambda_{\mathrm{D}}$. Nevertheless, we can solve in general Eq. (S38) numerically to check that the Debye-Hückel regime remains valid for the experimental data of inlet surface charges for various concentrations.

III. SUPPLEMENTARY NOTE S3: FINITE-ELEMENT CALCULATIONS

The goal of this section is to confirm the hypothesis and mechanisms of the model presented in Supplementary Note 2. Thus we focus on presenting a realistic geometry of the system and resolving the small charge-separated region at the activated inlet, which sets the concentration boundary conditions across the channel. On Supplementary Figure S12, we show the mesh used in the simulations and summarize the model's hypothesis.

A. Model

Two 5 nm long reservoirs are placed on both sides of the channel, on the walls of which the pressure and concentration boundary conditions are applied. The channel length L cannot be realistically taken as 5 μm and is fixed at 100 nm in all further calculations. This will lead to a much smaller Péclet number and thus a smaller amplitude of the mechanosensitive effect compared to experiments, as we discuss below. The channel walls have a perfect slip boundary condition and no surface charge $\Sigma_{\mathrm{ch}} = 0$. The charged inlet is modeled by imposing a surface charge density Σ_{inlet} on the top wall at the left inlet of the channel, very close to the experimental geometry where the plasma exposure activates this same region.

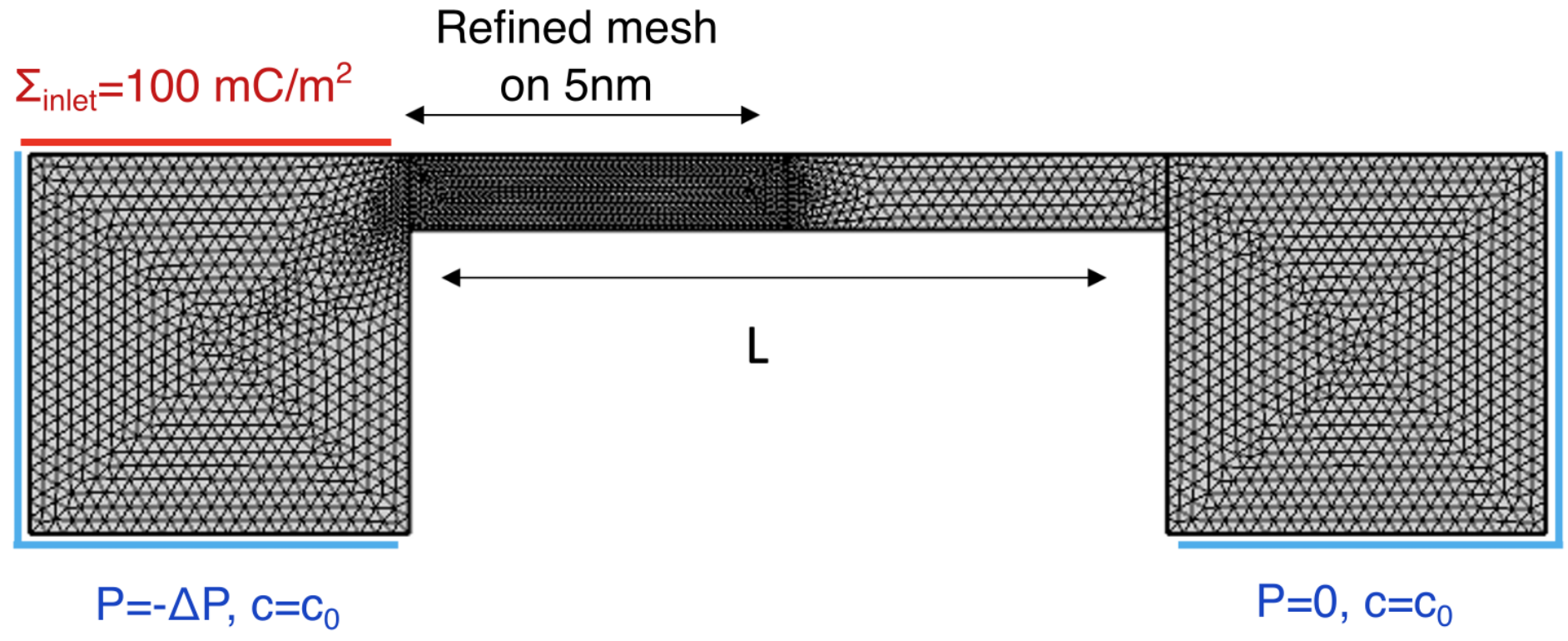

Supplementary Figure S12: **Comsol mesh** with $L = 100$ nm. Note here Σ_{inlet} (red line) is imposed on the top wall at the left inlet of the channel. Pressure and concentration boundary conditions are applied on the left and right reservoirs and perfect slip boundary conditions are imposed on the channel walls. The 5 nm region close to the transition between the inlet and channel regions has a strongly refined mesh. This allows to properly resolve the concentration and velocity fields at the channel inlet where gradients are very strong and where a nm-scale charge separation region develops. For long channels (we used typically L=100 nm), using such a fine mesh on the full channel length yields unreasonably heavy computations without any improvement of the results.

A pressure drop ΔP is imposed between the two reservoirs, while no voltage drop is applied across the channel ($\Delta V = 0$). Note the refined mesh on a 5 nm region close to the inlet. The bulk salt concentration c_0 is imposed in both reservoirs. The fluid flow, ionic transport and electrostatics are solved self-consistently using the coupled Poisson-Nernst-Planck and Stokes equations.

B. Concentration and fields in the inlet region

In Supplementary Figure S13, we zoom in the region close to the inlet and plot the horizontal (panel **b**) and vertical (on panel **d**) ion-concentration profiles across the few nanometers close to the inlet at $x = 0$. We also show the salt concentration profile along the channel length $c(x)$. This clearly shows that the charge separation does not extend over more than 1 nm into the channel along x. The concentration change in the inlet region is of the order of 10 percent (100 mM) in contrast with the 0.6 mM (0.06 percent) in the channel

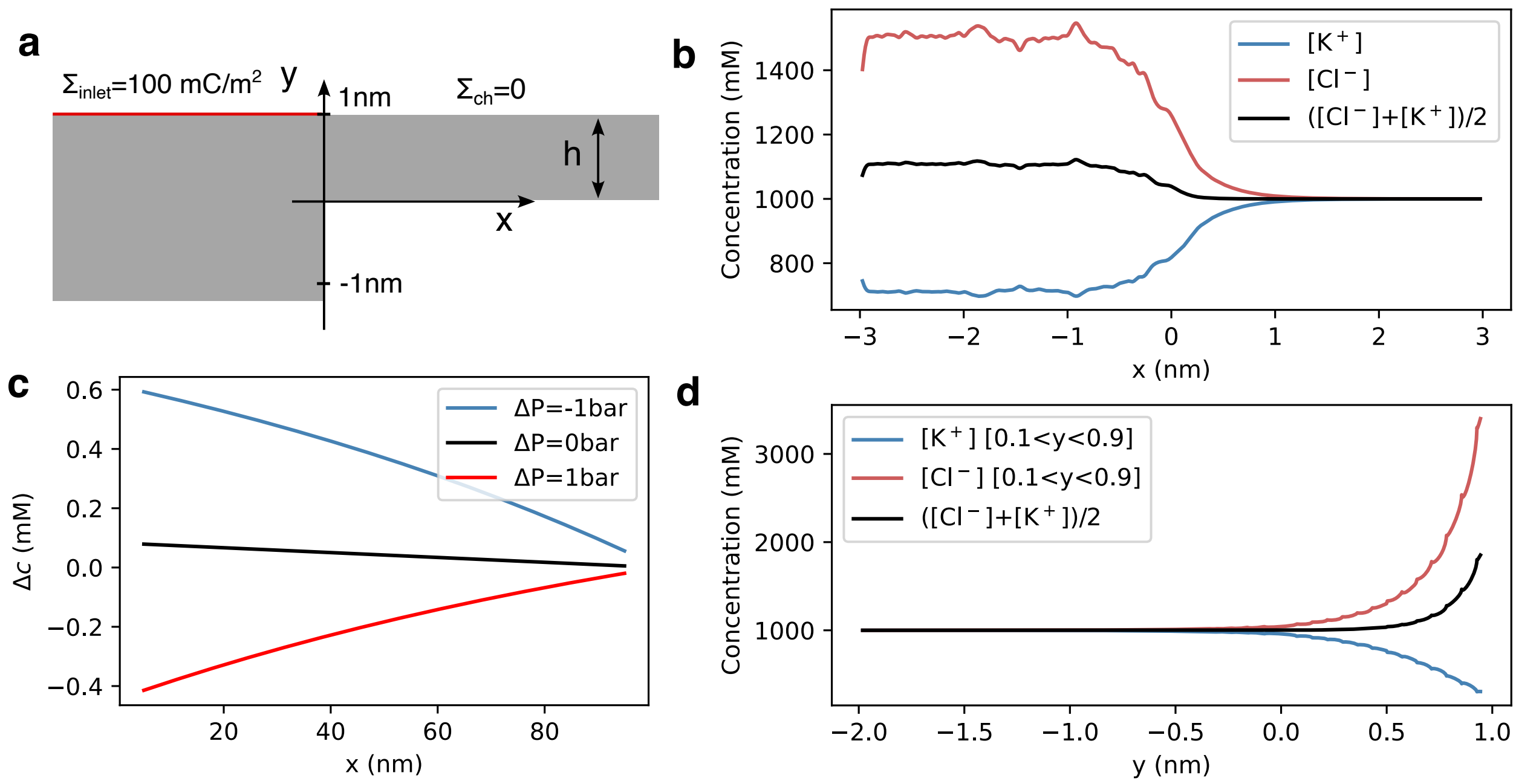

Supplementary Figure S13: **Concentration profiles from the finite element calculations** (**a**) Schematic of the geometry corresponding to the mesh shown on Supplementary Figure S12. (**b**) Horizontal concentration profile (averaged for $0 < y < 1$ nm) along a few nanometers around x=0. (**c**) Horizontal concentration change profile along the full channel length for pressure drops of 0, -1 and 1 bar. (**d**) Vertical concentration profile in the inlet region averaged for -1 nm$< x < 0$.

for a 1 bar pressure drop.

In Supplementary Figure S14, we further show colormaps of the electrical potential and charge density fields at zero pressure drop (panels **a-b**) as well as the velocity field and charge density under a -1 bar pressure drop (panel **c**). The charge separation region is clearly visible close to the charged inlet and the electrical potential map clearly shows the very small spatial extension of electric potential gradients along x, $\partial_x \phi$ which clearly vanish at sub-nanometer scales.

C. Mechanosensitive response

We extract the concentration profiles at various pressure drops (see Supplementary Figure S15 **a**). Note the very close similarity with the profiles computed from the analytical model in Figure 2**b** of the main text. From the profiles, we compute the relative conductance

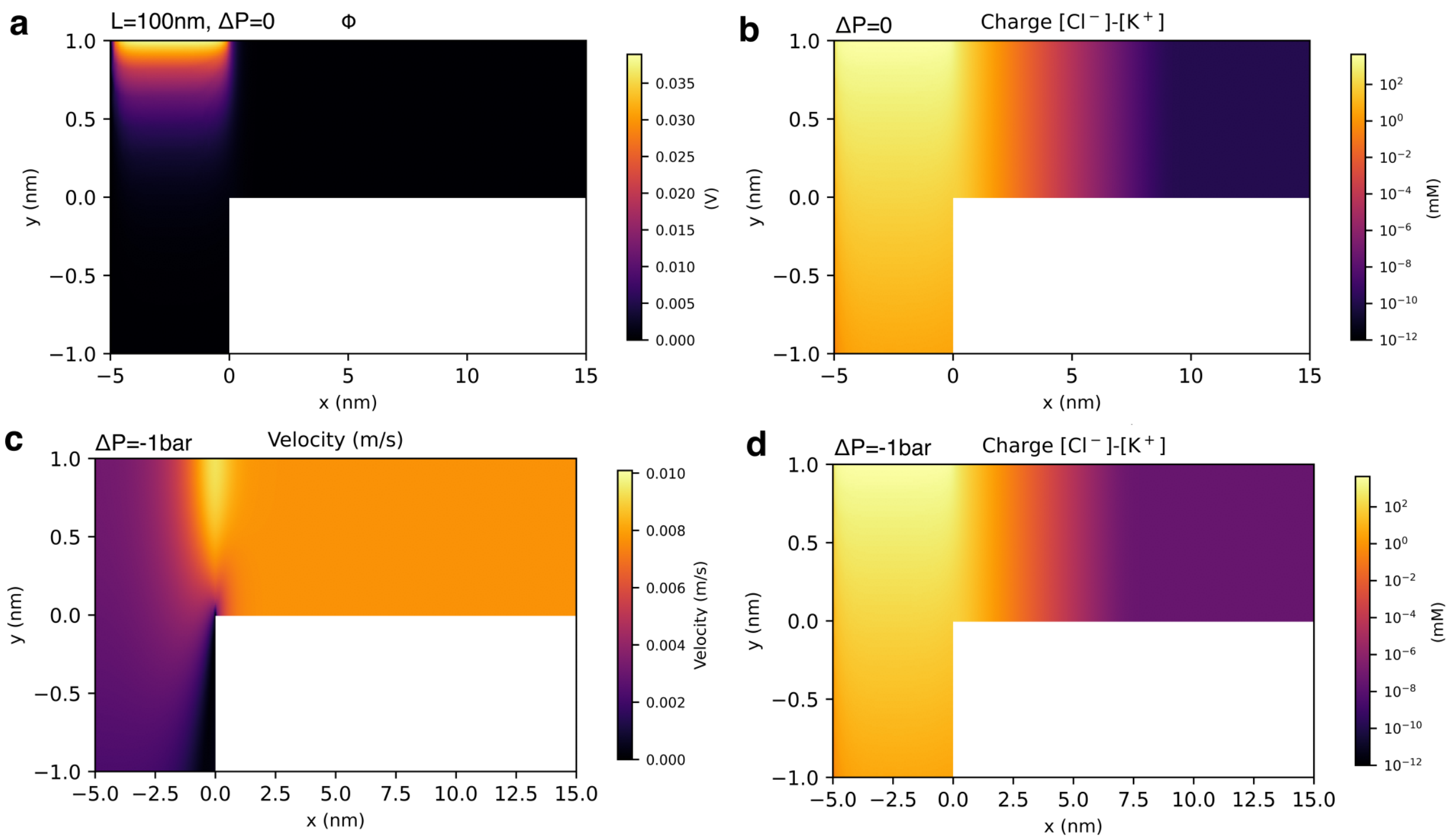

Supplementary Figure S14: **Concentration, potential and charge density fields from finite element calculations** (a) Colormaps for the electrical potential ϕ in V and (b) charge density in mM for L =100 nm, Σ_{inlet}=0.1 C/m^2 at zero pressure drop. (c) Velocity field and charge density for L =100 nm, Σ_{inlet}=0.1 C/m^2 under a -1 bar pressure drop.

change with the local Ohm's law and plot the relative mechanosensitivity on panel **b**. As in experiments, we also find a linear regime at low pressure drops, reproducing the odd mechanosensitive signature.

D. High pressure-drop regime

In contrast to experiments however, we find a strong asymmetry at high ΔP between positive and negative pressure drops. Interestingly, this asymmetry is also captured by the 1D non-linear model presented in Supplementary Note 2 (see the high pressure saturation Supplementary Figure S11 **c**).

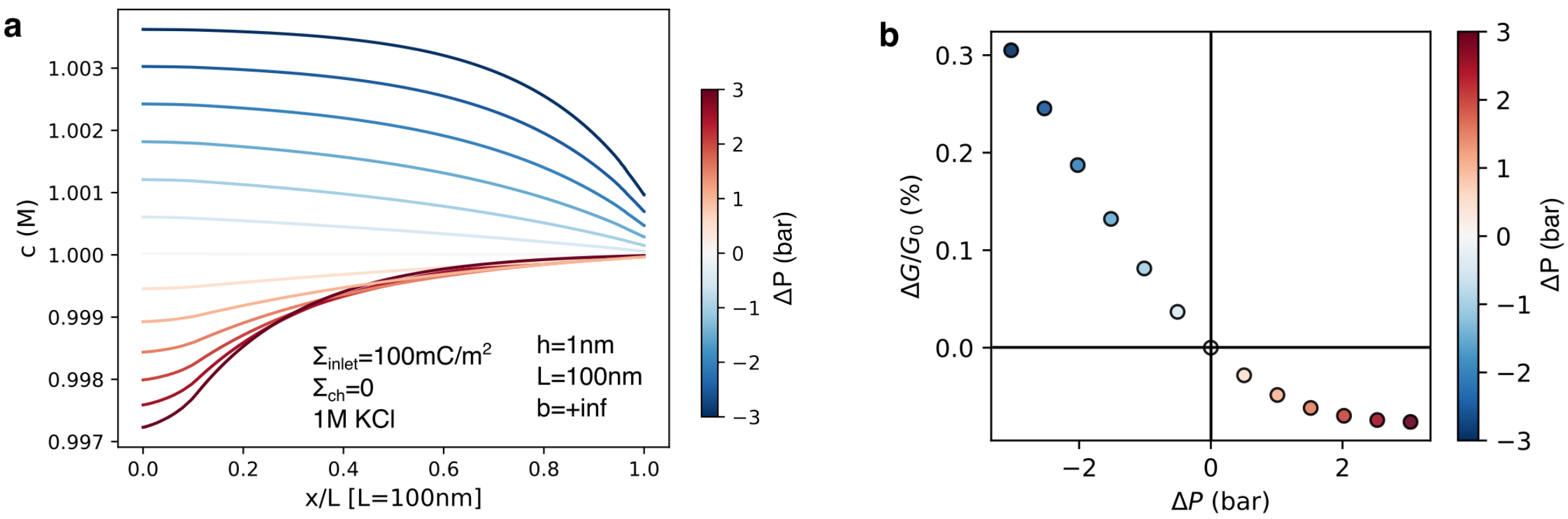

Supplementary Figure S15: **Pressure-dependence of conductance change** in the Comsol model. The concentration profiles are given on panel **a** for various pressure drops while the conductance change is given on panel **b**.

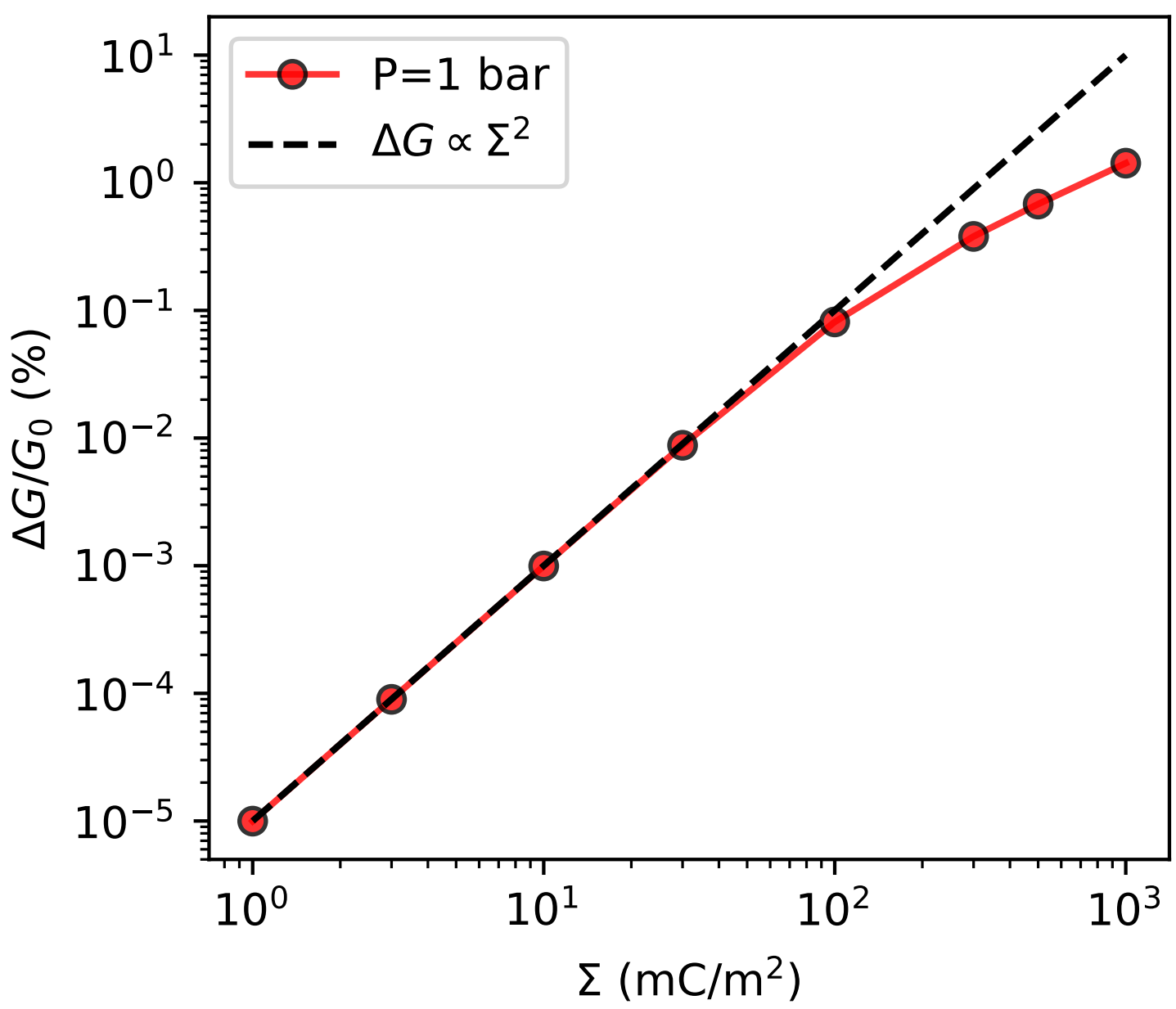

Supplementary Figure S16: Σ_{inlet}**-dependence of the relative mechanosensitivity** in the infinite slippage Comsol model. In the low Dukhin number limit, we find a scaling $\Delta G/G_0 \sim \Sigma_{\text{inlet}}^2$ predicted analytically in the main text.

E. Quantitative comparison

The smaller amplitude (roughly 0.1 % at 1 bar) is due to the smaller channel length ($L =$ 100 nm) versus $L = 5\ \mu$m in experiments. Since we expect $\Delta G/G_0 \propto \text{Pe}$, we anticipate

a reduction by a factor of 50 with respect to the experimental value $\Delta G/G_0 \sim 10\%$, in very good agreement with the results. This semi-quantitative matching further validates our interpretation and the quantitative model presented in Supplementary Note 2.

F. Surface charge scaling

On Supplementary Figure S16, we show the influence of surface charge Σ_{inlet} on the relative pressure-induced conductance change $\Delta G/G_0$. We find a power law $\Delta G/G_0 \sim \Sigma_{\text{inlet}}^2$ in excellent agreement with the analytical result for the high concentration limit (see Eq.11 of the main text). The deviation at high surface charge is expected since the scaling is derived in the low Dukhin regime whereas a weaker dependence is expected at high Dukhin number (Eq.10 of the main text).

G. Advection-diffusion problem

Let us now elaborate on the experimental results obtained with a pristine inlet and a concentration drop ΔC in Supplementary Figure S7. We confirm here that the problem can be reduced to that of a concentration drop across the channel. First, we use the Comsol simulations to extract the full concentration profile for a range of pressure drops (see Supplementary Figure S17**a**). From the profile, we extract the concentration imbalance ΔC at the charged inlet with respect to the bulk concentration c_0. Then, we use a simple 1d drift-diffusion model – neglecting diffusio-osmotic contribution – with concentration boundary conditions of $\Delta C + c_0$ and c_0:

$$c(x) - c_0 = \Delta C \frac{\exp(vx/D) - 1}{\exp(vL/D) - 1} \tag{S43}$$

We find that the resulting concentration profile and conductance change are very close to those extracted directly from the finite element solutions. The results are reported on Supplementary Figure S17 **b-c**.

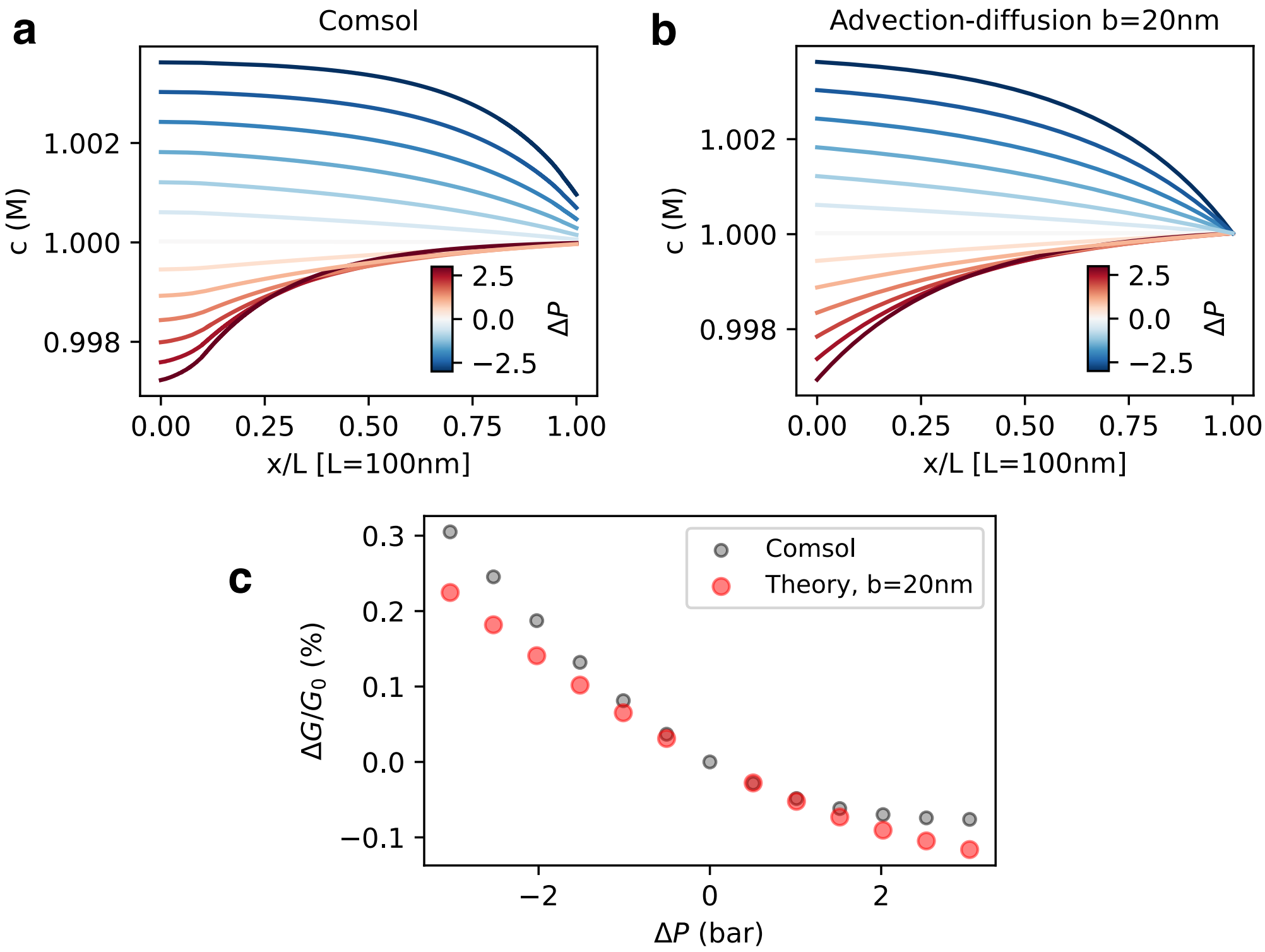

Supplementary Figure S17: **Advection-diffusion profile** from the Comsol-computed concentration drop. (**a**) Concentration profiles in the Comsol model for various pressure drops. (**b**) Concentration profile from the advection-diffusion equation with the same concentration drop at the inlet as in the Comsol solution. (**c**) Comparison of the relative conductance change from Comsol and from the advection-diffusion profiles with the same concentration drop at the inlet.
